# A positive feedback inhibition of isocitrate dehydrogenase 3β on paired-box gene 6 promotes Alzheimer-like pathology

**DOI:** 10.1038/s41392-024-01812-5

**Published:** 2024-04-29

**Authors:** Xin Wang, Qian Liu, Hai-tao Yu, Jia-zhao Xie, Jun-ning Zhao, Zhi-ting Fang, Min Qu, Yao Zhang, Ying Yang, Jian-Zhi Wang

**Affiliations:** 1 Department of Pathophysiology, School of Basic Medicine, Key Laboratory of Education Ministry of China/Hubei Province for Neurological Disorders, Tongji Medical College, Huazhong University of Science and Technology, Wuhan, China; 2 Department of Fundamental Medicine, Wuxi School of Medicine, Jiangnan University, Wuxi, Jiangsu 214122 China; 3 Hubei Provincial Key Laboratory for Applied Toxicology, Hubei Provincial Center for Disease Control and Prevention, Hubei Provincial Academy of Preventive Medicine, Wuhan, 430000 China; 4 Endocrine Department of Liyuan Hospital; Key Laboratory of Ministry of Education of China for Neurological Disorders, Tongji Medical College, Huazhong University of Science and Technology, Wuhan, 430077 China; 5 Co-innovation Center of Neuroregeneration, Nantong University, Nantong, 226000 China

**Keywords:** Diseases of the nervous system, Metabolic disorders

## Abstract

Impaired brain glucose metabolism is an early indicator of Alzheimer’s disease (AD); however, the fundamental mechanism is unknown. In this study, we found a substantial decline in isocitrate dehydrogenase 3β (IDH3β) levels, a critical tricarboxylic acid cycle enzyme, in AD patients and AD-transgenic mice’s brains. Further investigations demonstrated that the knockdown of IDH3β induced oxidation-phosphorylation uncoupling, leading to reduced energy metabolism and lactate accumulation. The resulting increased lactate, a source of lactyl, was found to promote histone lactylation, thereby enhancing the expression of paired-box gene 6 (PAX6). As an inhibitory transcription factor of IDH3β, the elevated PAX6 in turn inhibited the expression of IDH3β, leading to tau hyperphosphorylation, synapse impairment, and learning and memory deficits resembling those seen in AD. In AD-transgenic mice, upregulating IDH3β and downregulating PAX6 were found to improve cognitive functioning and reverse AD-like pathologies. Collectively, our data suggest that impaired oxidative phosphorylation accelerates AD progression via a positive feedback inhibition loop of IDH3β-lactate-PAX6-IDH3β. Breaking this loop by upregulating IDH3β or downregulating PAX6 attenuates AD neurodegeneration and cognitive impairments.

## Introduction

Alzheimer’s disease (AD) is a widespread degenerative neurological disease with a fast-growing prevalence in the elderly, imposing a huge economic and emotional burden on individuals, families, and society.^1^ The development of tau neurofibrillary tangles and amyloid-β (Aβ) plaques are two of the primary pathological characteristics of AD.^2–7^ Recent Aβ immunotherapies have bolstered support for the Aβ hypothesis and expedited approval.^8–10^ While these therapies have led to notable reductions in fibrillar amyloid plaque pathology, their impact on cognitive and functional outcomes remains modest. To achieve substantial clinical benefits, a comprehensive and multifaceted approach may be necessary.

The brain uses glucose as its primary energy source due to its high energy metabolism.^11,12^ During the pathogenesis of AD, age-dependent reductions in brain glucose metabolism occur initially in areas closely associated with memory, preceding cognitive dysfunction and AD-related changes.^12–15^ Reduced glucose metabolism leads to reduced ATP biosynthesis, which in turn leads to a reduction in the ability of neurons to maintain ionic gradients and impedes action potential production. This disruption of ionic gradients results in an influx of extracellular Ca^2+^, leading to mitochondrial dysfunction, neuronal apoptosis, and AD-like pathology.^12,16–18^ These findings underscore the critical role of glucose metabolism disorders in AD development.

Mitochondria are involved in aerobic glucose oxidation, which includes the tricarboxylic acid cycle (TCA) and respiratory chain oxidative phosphorylation. Studies have reported that glucose metabolism disorders in AD patients primarily affect the TCA segment.^19^ In the TCA cycle, isocitrate dehydrogenase 3 (IDH3) is a rate-limiting enzyme that converts NAD^+^ to NADH and oxidatively decarboxylates isocitrate into α-ketoglutarate and CO_2_. The respiratory chain receives protons through this process, which facilitates energy supply and oxidative phosphorylation.^20,21^ In the pathological progression of AD, IDH3 activity is reduced by 27%.^22^ IDH3 comprises two α catalytic subunits, a β structural subunit, and a γ metathesis subunit. Its monomeric state is inactive, and full activity is achieved when it forms a heterotetramer in a 2:1:1 ratio. IDH3β is crucial for heterotetramer assembly. It is widely distributed in vivo and can be activated by ADP, NAD^+^, Mg^2+^, and isocitric acid metathesis during tetramer formation, while ATP and NADH inhibit its activity.^23,24^ These findings highlight the significance of IDH3β in energy metabolism.

Histone post-translational modifications (PTM) regulate several essential biological functions.^25,26^ Various metabolites lead to histone post-translational modifications, such as β-hydroxybutyrylation,^27^ crotonylation,^28^ and succinylation.^29,30^ Histone PTMs affect genome function directly and indirectly.^31^ Recent studies have shown that lactate, a product of glycolysis, can induce histone lactylation.^32^ AD mice undergo metabolic remodeling from oxidative phosphorylation to glycolysis,^33^ and brain histone lactylation in the 5xFAD mouse model increases glycolytic gene transcripts and activity.^34^

This study found attenuated IDH3β expression in the brains of patients with AD. Utilizing siRNA technology, we conducted knockdown experiments targeting IDH3β, which resulted in a decline in TCA cycle rate, ATP production, and intracellular lactate accumulation. Additionally, we observed elevated levels of histone lactylation and increased expression of transcription factors such as PAX6. Increased PAX6 expression inhibits IDH3β through positive feedback, causing synaptic damage and impaired learning and memory. In contrast, both upregulation of IDH3β and downregulation of PAX6 improved synaptic function and mitigated learning and memory deficits by enhancing cellular energy metabolism and reducing histone lactylation. Our study sheds light on the IDH3β-lactate-PAX6-IDH3β positive feedback mechanism that underlies metabolic disorders, emphasizing the modulating potential of IDH3β as a new molecular target for AD therapy.

## Results

### Both AD-transgenic mice and AD patients show a considerable drop in IDH3β levels in their brains

**Fig. 1:**
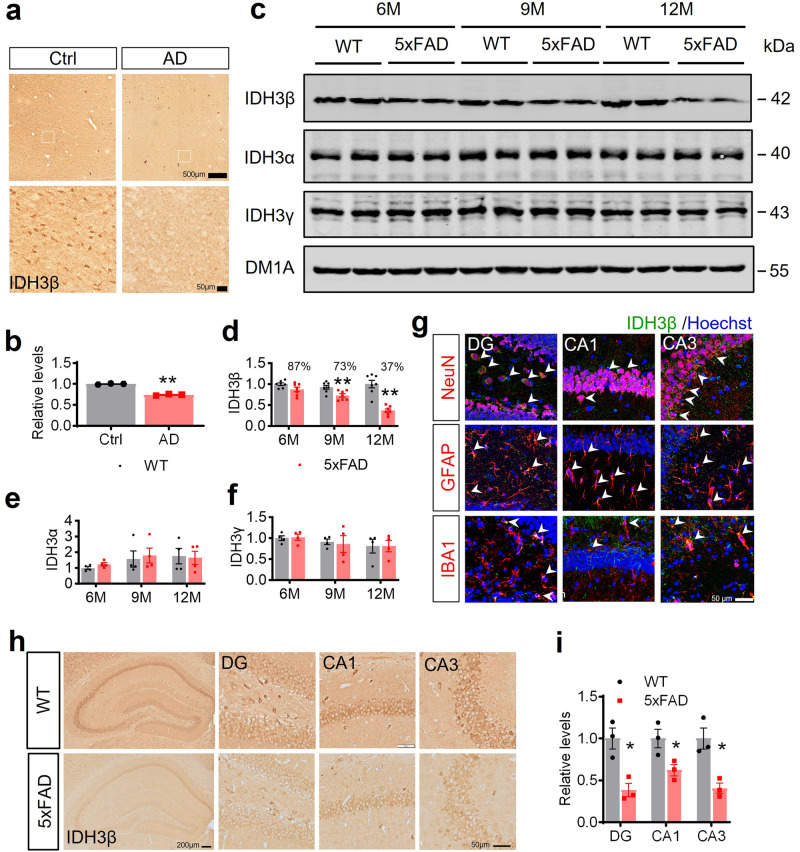
IDH3β protein levels are decreased in the brain of AD patients and the hippocampus of AD-transgenic mice. **a**, **b** Immunohistochemistry staining reveals an IDH3β reduction in the HP of patients with AD at stage III in comparison to a healthy control group. Whole bar = 500 µm, area bar = 50 µm. For each group (n = 3); Two-tailed Student’s t-test; **P < 0.001. **c**–**f** Western blotting revealed an age-dependent decrease in the level of IDH3β protein in the HP of 5xFAD mice, whereas no such decline was observed in the littermates. The levels of IDH3α and IDH3γ proteins did not differ significantly from the control group. IDH3β, 6 m, *P* = 0.067; 9 m, **P = 0.0099; 12 m, **P < 0.001; for each group (n = 7); IDH3γ and IDH3γ, for each group (n = 4); Two-tailed Student’s t-test. **g** IDH3β was co-localized with neurons (NeuN), astrocytes (GFAP), and microglia (IBA1) and measured using immunofluorescence staining. Scale bar, 50 µm. **h**, **i** Immunohistochemistry staining revealed a decrease in IDH3β levels in the hippocampal subsets of 12-month-old 5xFAD mice in comparison to the control group. Area bar = 50 µm; total HP, bar = 200 µm. DG, *P = 0.014; CA1, *P = 0.044; CA3, *P = 0.013; for each group (n = 3); Two-tailed Student’s t-test. The format of mean ± SEM was utilized to display the data

To examine alterations in IDH3β during the AD process, we conducted immunostaining on brain sections from patients with AD and AD-transgenic mice. AD brains exhibited considerably lower IDH3β signals as compared with healthy controls (Fig. 1a, b). Human APP and PSEN1 transgenes harboring five AD-associated mutations are utilized in the 5xFAD transgenic mouse model, which is an established model of AD. Western blotting analysis conducted on 5xFAD mice demonstrated a significant age-dependent reduction in IDH3β, with statistical significance being reached at 9 and 12 months (Fig. 1c, d). However, protein levels of IDH3α and IDH3γ did not differ significantly from those of the control (Fig. 1e, f). To investigate the distribution of IDH3β among different types of neuronal cells, we conducted double immunostaining using distinct markers such as NeuN, GFAP, and IBA1 for neurons, astrocytes, and microglia cells, respectively. Colocalization of IDH3β signals with NeuN, GFAP, and IBA1 was observed across all subregions of the hippocampus (HP), suggesting that IDH3β exhibited extensive expression across all neural cells (Fig. 1g). In addition, compared with control mice, IDH3β signals within pyramidal neurons and granule cells were remarkably diminished in 12-month-old 5xFAD mice (Fig. 1h, i).

### Downregulating IDH3β induces cognitive deficits with impaired energy metabolism

**Fig. 2:**
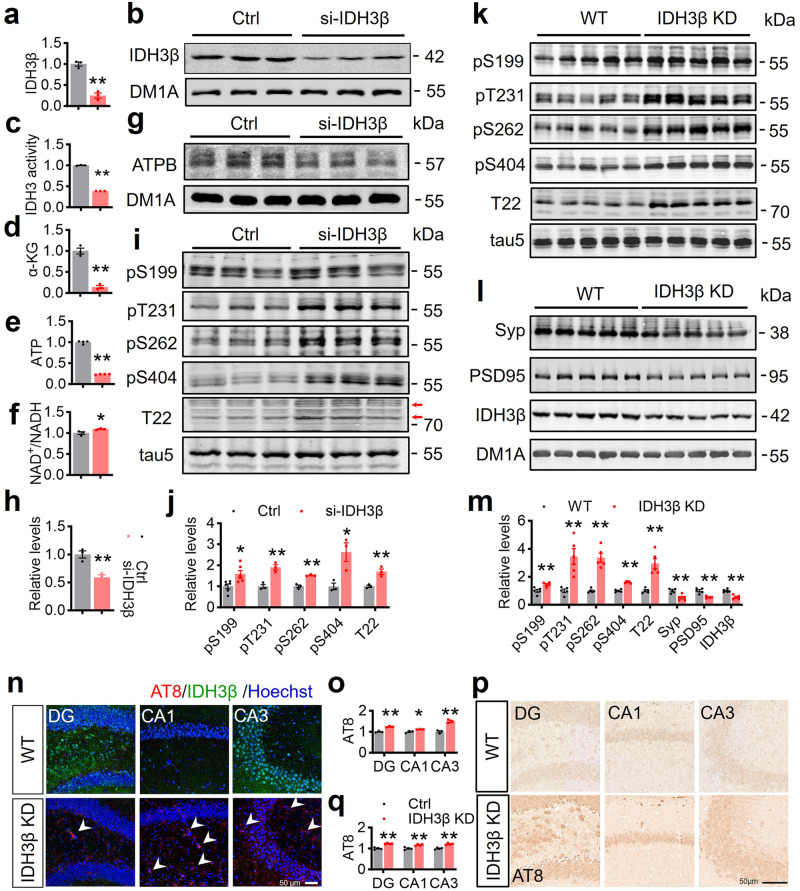
Downregulating IDH3β induces AD-like pathologies with impaired synaptic plasticity and energy metabolism. **a**–**c** Application of siRNA induced 75% reduction of IDH3β protein level with 62% reduced IDH3β enzyme activity in N2a cells. For each group (n = 3); *P < 0.001, Two-tailed Student’s t-test. **d**–**f** Knockdown of IDH3β decreased levels of α-ketoglutarate and ATP with an increased ratio of NAD^+^ to NADH in N2a cells. Two-tailed Student’s t-test, d, for each group (n = 3); **P < 0.001; e, for each group (n = 4); **P < 0.001; f, for each group (n = 3); *P = 0.045. **g**, **h** Knockdown of IDH3β decreased the level of ATP synthase β-subunit (ATPB) in N2a cells. For each group (n = 3); **P = 0.006, Two-tailed Student’s t-test. **i**, **j** Knockdown of IDH3β significantly increased the phosphorylation levels of tau at S199 (*P = 0.013, n = 6), T231 (**P = 0.005, n = 3), S262 (**P = 0.002, n = 3), S404 (*P = 0.025, n = 3) and T22-positive tau (the oligomer tau, the main band with an arrow) (**P = 0.008, n = 3) in N2a cells. Two-tailed Student’s t-test. **k**–**m** Knockdown of IDH3β increased the phosphorylation levels of tau at S199 (**P = 0.006), T231 (**P = 0.003), S262 (**P < 0.001), S404 (**P < 0.001) and T22-positive tau (**P < 0.001) and decreased level of synaptophysin (Syp) (**P = 0.003) and postsynaptic density protein 95 (PSD95) (**P < 0.001) measured using Western blotting in C57BL/6 mice aged 2 months. For each group (n = 5); IDH3β, **P < 0.001; Two-tailed Student’s t-test. **n**–**q** Knockdown of IDH3β increased the level of AT8 (pTau) measured using immunofluorescence staining (**n**, **o**) and immunohistochemistry staining (**p**, **q**) in C57BL/6 mice aged 2 months. Scale bar, 50 µm. Two-tailed Student’s t-test, (**n**, **o**), DG, **P < 0.001, CA1, *P = 0.023, CA3, **P = 0.002; for each group, (n = 3); **p**, **q** DG and CA3, **P < 0.001, CA1, **P = 0.003; for each group (n = 4). The format of mean ± SEM was utilized to display the data

IDH3 is a rate-limiting enzyme of the TCA cycle. To investigate the effects of diminished IDH3β levels on energy metabolism, we employed siRNA to knock down IDH3β expression in N2a cells. A 75% decrease in IDH3β protein levels (Fig. 2a, b) and a 62% decline in IDH3β enzyme activity (Fig. 2c) were observed. This reduction was accompanied by a decrease in α-ketoglutarate (α-KG), an intermediate product of the TCA cycle (Fig. 2d), as well as reduced ATP production (Fig. 2e) and an increased NAD^+^ to NADH ratio (Fig. 2f). ATP production is primarily driven by the electron respiratory chain, which comprises of five enzyme complexes and mobile electron carriers.^20^ Upon further examination of the protein levels of these enzyme complexes, we found that the β-subunit of ATP synthase was reduced, whereas NADUFB10 and NDUFS1 of respiratory chain complex I, SDHB of complex II, UQCRFS1/ RISP of complex III, α subunit of ATP synthase, and voltage-dependent anion channel, VDAC 1 and 3 did not show significant changes (Fig. 2g, h, Supplementary Fig. s1a, b). These data demonstrate a disruption in oxidative phosphorylation resulting from reduced IDH3β levels, indicating a strong association between impaired energy metabolism mediated by IDH3β and the progression of AD.

Phosphorylated tau (pTau) accumulation is a hallmark cause of AD.^7,35^ Results of Western blot analysis revealed that IDH3β knockdown in N2a cells significantly elevated tau phosphorylation at S199, T231, S262, S404 and T22-positive tau (the oligomer tau) (Fig. 2i, j). Supplementing with α-KG notably attenuated these AD-like tau pathologies (Supplementary Fig. s2a, b). To study the impact of decreased IDH3β on AD-like pathogenesis in vivo, we generated IDH3β knockdown transgenic mice (IDH3β KD) and examined tau pathology and synaptic protein changes. IDH3β knockdown in 2-month-old mice substantially enhanced tau phosphorylation levels at S199, T231, S262, S404, and T22-positive tau, supporting our cellular findings. (Fig. 2k). Western blot analysis findings additionally demonstrated a reduction in synaptophysin (Syp) and postsynaptic density protein 95 (PSD95) levels following IDH3β knockdown (Fig. 2l, m). The absence of AT8 (pTau)+ signals was significantly greater in the IDH3β KD group as determined using immunostaining, in contrast to the wild-type group (Fig. 2n–q). Findings suggest that suppressing IDH3β leads to AD-like pathologies.

**Fig. 3:**
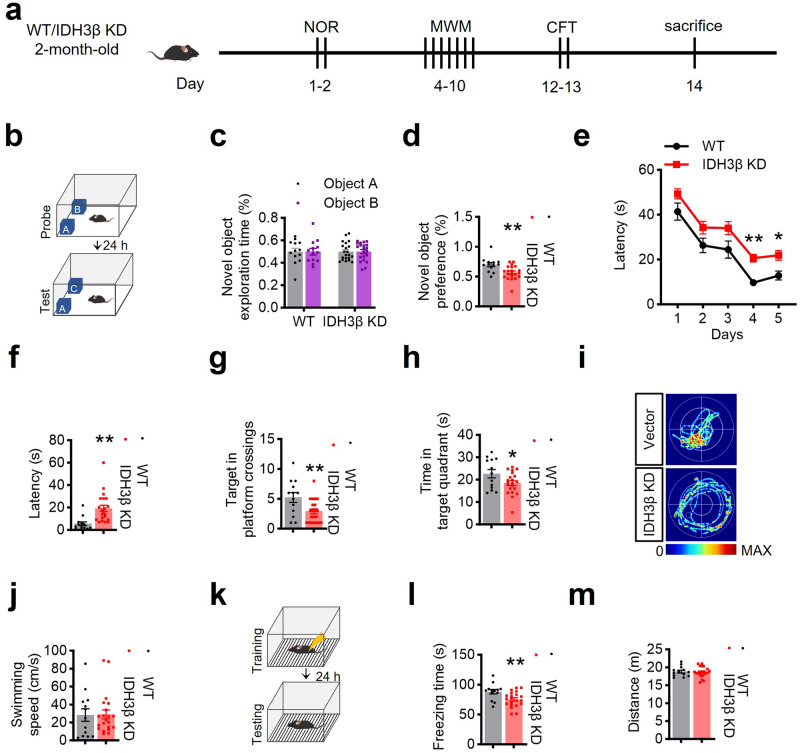
Downregulating IDH3β induces cognitive deficits. **a** Behavioral tests were performed for cognitive function after knockdown of IDH3β in 2-month-old C57BL/6 mice: novel object recognition test (NOR) for spatial memory, Morris water maze test (MWM) for spatial learning and memory, and contextual fear condition test (CFT) for contextual fear memory. **b**–**d** knockdown of IDH3β displayed comparable exploration time during the sample phase and exhibited decreased novelty preference in 2-month-old C57BL/6 mice in NOR. Two-tailed Student’s t-test, **P = 0.003, WT, n = 13 mice, IDH3β KD, n = 21 mice. **e**–**j** IDH3β knockdown impaired spatial learning and memory in C57BL/6 mice aged 2 months, as evidenced by increased latency during 5-day training trials in MWM, decreased target platform crossings, and decreased time spent in the target quadrant during probe test on day 7. Swimming speed did not differ between the two groups. **e** Two-way repeated measures ANOVA test followed by the Bonferroni’s post hoc test, day 4, **P < 0.001, day 5, *P = 0.014; **f** Two-tailed Student’s t-test, **P = 0.001; **g** Two-tailed Student’s t-test, **P = 0.009; **h** Two-tailed Student’s t-test, *P = 0.045; WT, n = 13 mice; IDH3β KD, n = 21 mice. **k**–**m** Knockdown of IDH3β impaired contextual fear memory measured using CFT evidenced by the decreased freezing time in 2-month-old C57BL/6 mice. The mice did not show any difference in movement distance. Two-tailed Student’s t-test, **P = 0.009, WT, n = 13 mice, IDH3β KD, n = 21 mice. The format of mean ± SEM was utilized to display the data

Next, we conducted a series of behavior tests to assess cognitive capacity in the presence of IDH3β reduction (Fig. 3a). Compared with their littermates, mice with IDH3β knockdown displayed comparable exploration time during the sample phase and exhibited decreased novelty preference in the novel object recognition test (NOR) (Fig. 3b–d). During 5-day training trials in the Morris water maze test (MWM), mice with IDH3β knockdown displayed prolonged latency in finding the target platform (Fig. 3e). Day 7’s probe test showed that IDH3β knockdown increased the latency to locate the target platform (Fig. 3f), decreased the number of target platform crossings (Fig. 3g) and the time spent in the target quadrant (Fig. 3h). The swim speeds of the two groups were identical (Fig. 3i, j). Additionally, we observed impaired memory in the contextual fear condition test (CFT), evidenced by decreased freezing time in the context (Fig. 3k, l). The distance traveled by the two groups did not differ significantly (Fig. 3m). These findings show that IDH3β knockdown negatively impacts spatial learning and memory.

### IDH3β reduction forms a positive feedback inhibition loop of IDH3β-lactate-PAX6-IDH3β by promoting histone lactylation

**Fig. 4:**
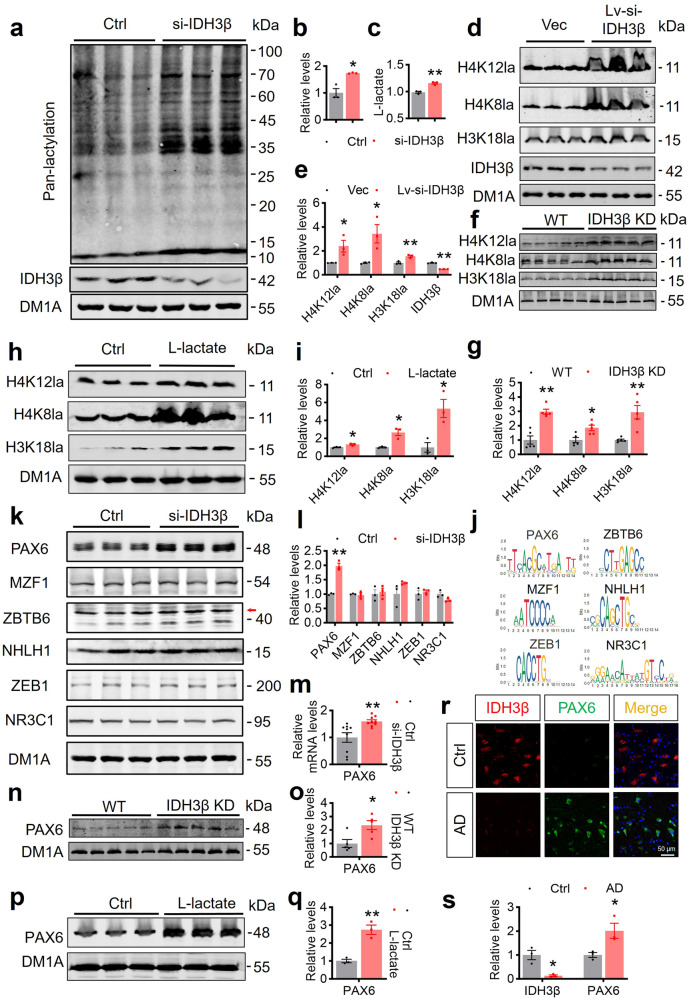
Downregulating IDH3β increases histone lactylation and promotes PAX6 expression. **a**–**c** Knockdown of IDH3β resulted in an increased lactate level with an increased protein pan-lactylation in N2a cells. For each group (n = 3); **b** *P = 0.01; **c** **P = 0.002; Two-tailed Student’s t-test. **d**, **e** Knockdown of IDH3β increased histone H4 lactylation at Lys12 (*P = 0.046) and Lys8 (*P = 0.034) and H3 at Lys18 (**P = 0.009) in primary neurons. For each group (n = 3); IDH3β; **P < 0.001; Two-tailed Student’s t-test. **f**, **g** Knockdown of IDH3β increased histone H4 lactylation at Lys12 (**P < 0.001) and Lys8 (*P = 0.012) and H3 at Lys18 (**P = 0.003) in the hippocampus of C57BL/6 mice aged 2 months. For each group (n = 5); Two-tailed Student’s t-test. **h**, **i** L-lactate (sodium lactate) (20 mM for 24 h) treatment increased histone H4 lactylation at Lys12 (*P = 0.019) and Lys8 (*P = 0.0104), and H3 at Lys18 (*P = 0.019) in N2a cells. For each group (n = 3); Two-tailed Student’s t-test. **j** Top transcription factor binding sites using GeneCards and JARPAR public databases in the IDH3β gene promoter are PAX6, MZF1, ZBTB6, NHLH1, ZEB1, and NR3C1. **k**–**m** Knockdown of IDH3β significantly increased protein and mRNA levels of PAX6 in N2a cells. the protein expression levels of MZF1, ZBTB6 (the main band with an arrow), NHLH1, ZEB1 and NR3C1 were not significantly different from the control. For each group (n = 3), **P < 0.001, Two-tailed Student’s t-test; **m** For each group (n = 9), **P = 0.006. **n**, **o** Knockdown of IDH3β significantly increased protein levels of PAX6 in the hippocampus of C57BL/6 mice aged 2 months. For each group (n = 5), *P = 0.016, Two-tailed Student’s t-test. **p**, **q** L-lactate (sodium lactate) (20 mM for 24 h) treatment increased PAX6 expression in N2a cells. For each group (n = 3), *P = 0.003, Two-tailed Student’s t-test. **r**, **s** A significant decrease in IDH3β levels and a notable increase in PAX6 levels in the hippocampus of patients with AD compared to the control group measured by immunofluorescence staining. Scale bar, 50 µm. For each group (n = 3), IDH3β, *P = 0.012, PAX6, *P = 0.037, Two-tailed Student’s t-test. The format of mean ± SEM was utilized to display the data

To explore the impact of IDH3β downregulation on lactate production and non-metabolic functions of lactate, we initially assessed the levels of lactate and protein lactylation in N2a cells following IDH3β knockdown. Downregulating IDH3β led to a remarkable rise in L-lactate levels and lactylation of entire proteins in N2a cells (Fig. 4a–c). Specifically, there was a notable elevation in lactylation levels of histone H4 at Lys12, Lys8, as well as H3 at Lys18 sites, both in primary neurons (Fig. 4d, e) and the HP after IDH3β knockdown (Fig. 4f, g). Previous studies have suggested that histones can undergo hyper-acetylation through acetyl-CoA when TCA slows down.^32^ However, we examined the acetylation of histones H3 and H4 at multiple sites (Acetyl-Histone H3 antibody recognizes acetylation at Lys9, Lys14, Lys18, Lys23, Lys27, while Acetyl-Histone H4 antibody detects acetylation at Lys5, Lys8, Lys12, Lys16), but did not observe any changes following IDH3β knockdown (Supplementary Fig. s3).

To determine whether these changes were due to elevated lactate, we treated N2a cells with sodium lactate and assessed the mRNA levels of IDH3β. Interestingly, we observed a significant reduction in IDH3β mRNA levels to ~50% of the control level upon sodium lactate treatment (Supplementary Fig. s4). Next, we examined histone lactylation levels after directly treating the cells with sodium lactate. We observed elevated levels of histone lactylation H4 at Lys12, Lys8, and H3 at Lys18 sites (Fig. 4h, i).

Histone lactylation has been shown to regulate gene transcription levels.^32,34^ To explore the regulation of histone lactylation on the transcription levels of IDH3β, we examined the changes in transcription factor for IDH3β. PAX6, MZF1, ZBTB6, NHLH1, ZEB1, and NR3C1 were identified as top transcription factor binding sites in the IDH3β gene promoter based on public database analysis^36^ (Fig. 4j). Downregulating of IDH3β elevated PAX6 protein levels with no differences in the levels of other transcription factors (Fig. 4k, l). Additionally, we observed an increase in PAX6 mRNA levels (Fig. 4m). After IDH3β knockdown, 2-month-old C57BL/6 mice exhibited elevated PAX6 protein levels in their HP (Fig. 4n, o). When directly treating cells with sodium lactate, we also observed elevated PAX6 expression (Fig. 4p, q). Additionally, we conducted an analysis of PAX6 protein levels in 5xFAD transgenic mice aged 6, 9, and 12 months and discovered that PAX6 protein levels increased significantly with age, peaking at 12 months. (Supplementary Fig. s5). PAX6 expression was significantly elevated in the entorhinal cortex, frontal cortex, HP, and temporal cortex of patients with AD based on a public database^37^ (Supplementary Fig. s6). Finally, we conducted co-staining of IDH3β and PAX6 in AD brain sections and observed a significant reduction in IDH3β+ signals and a robust increase in PAX6+ signals in an AD patient’s brain compared to healthy controls (Fig. 4r, s), indicating a strong negative correlation between these two proteins in vivo. Together, these data suggest that downregulating IDH3β leads to elevated histone lactylation through elevated lactate, which promotes PAX6 transcription factor expression.

**Fig. 5:**
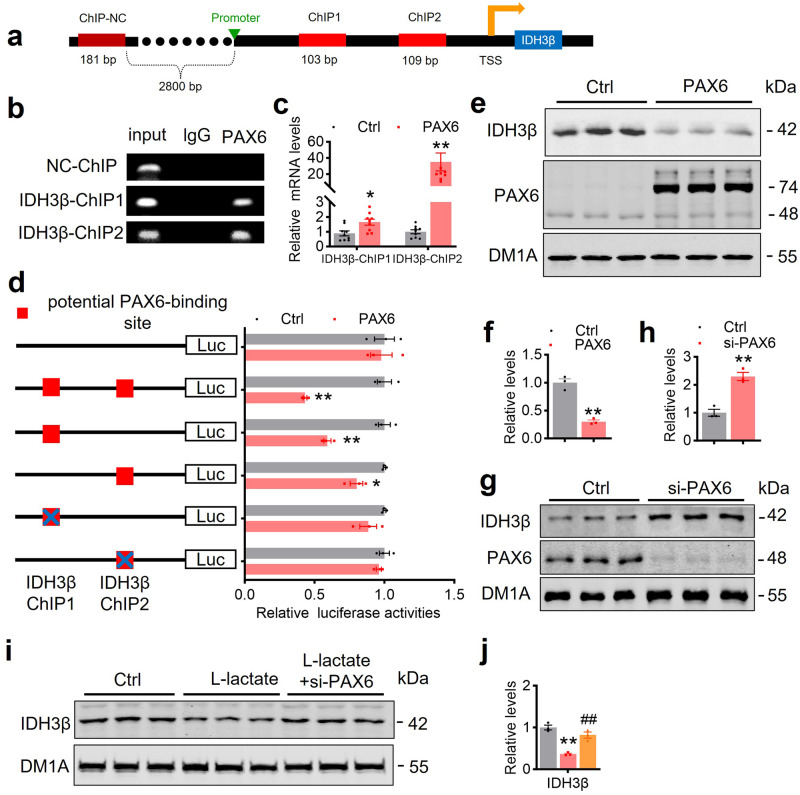
Upregulating PAX6 in turn inhibits IDH3β expression. **a**, **b** The direct binding of PAX6 to the IDH3β promoter was confirmed using ChIP assay in N2a cells, encompassing nonspecific control (NC) (a sequence located 2800 bp upstream from the transcription start site), IDH3β-ChIP1, and IDH3β-ChIP2. **c** In N2a cells, the direct binding capacity of PAX6 to the IDH3β promoter was validated using RT-PCR of the ChIP products. ChIP 1, *P = 0.011, ChIP 2, **P = 0.007, Two-tailed Student’s t-test; for each group (n = 9). **d** Binding PAX6 to IDH3β inhibited the transcription of IDH3β in a ChIP 1 and ChIP 2 element-dependent manner detected in N2a cells by dual-luciferase reporter gene assay, and both ChIP 1 and ChIP 2 mutations abolished the inhibition. ChIP 1, **P = 0.0012, ChIP 2, *P = 0.012, both, **P < 0.001; for each group (n = 3); Two-tailed Student’s t-test. **e**–**h** Overexpressing PAX6 inhibited IDH3β expression while knockdown PAX6 promoted IDH3β expression in N2a cells. **f** **P = 0.003; h, **P < 0.001; for each group (n = 3); Two-tailed Student’s t-test. **i**, **j** L-lactate (sodium lactate) (20 mM for 24 h) treatment significantly decreased protein levels of IDH3β, this decrease was completely reversed after PAX6 knockdown in N2a cells. **P < 0.001 *vs* Ctrl, ##*P* = 0.003 *vs* L-lactate; for each group (n = 3); One-way ANOVA test followed by Tukey’s post hoc test. The format of mean ± SEM was utilized to display the data

Expression of PAX6 is ubiquitous within the central nervous system.^38^ Overexpression of PAX6 causes abnormal ocular neurodevelopment.^39^ However, the regulation of IDH3β expression by PAX6 has not been reported. Through public database analysis,^40^ we found that PAX6 has two binding sequences in the IDH3β gene promoter region, namely ChIP 1 and ChIP 2, we chose a sequence 2800 bp upstream from the transcription start site as a nonspecific control (NC) (Fig. 5a). Then, we confirmed the direct binding of PAX6 to IDH3β by ChIP assay and RT-PCR in N2a cells (Fig. 5b, c). It is known that PAX6 possesses both transcriptional activation and repression functions.^41^ We created ChIP 1, ChIP 2, and the mutant sequences, which we then inserted either jointly or separately into luciferase reporter gene plasmids to ascertain the regulation of IDH3β expression by PAX6. The dual-luciferase reporter gene assay results revealed that binding PAX6 to IDH3β inhibited the transcription of IDH3β in a ChIP 1 and ChIP 2 element-dependent manner, and both ChIP 1 and ChIP 2 mutations abolished the inhibition (Fig. 5d). Furthermore, overexpressing PAX6 inhibited IDH3β expression, while knocking down PAX6 promoted IDH3β expression as measured by Western blotting in N2a cells (Fig. 5e–h). These results suggest that PAX6 inhibits IDH3β expression by binding to the ChIP 1 and ChIP 2 sequences in the IDH3β gene promoter. To identify whether PAX6 increases lactate-decreased IDH3β, we simultaneously treated N2a cells with sodium lactate and knocked down PAX6. In the lactate-treated group, IDH3β protein and mRNA levels decreased significantly but were completely restored following PAX6 knockdown (Fig. 5i, j, Supplementary Fig. s3).

Therefore, the above results suggest that reduced IDH3β expression can increase histone lactylation, promoting PAX6 expression. In turn, the elevated PAX6 exacerbates the reduction of IDH3β through positive feedback regulation.

### Upregulating IDH3β ameliorates AD-like pathologies and memory deficits in 5xFAD mice

**Fig. 6:**
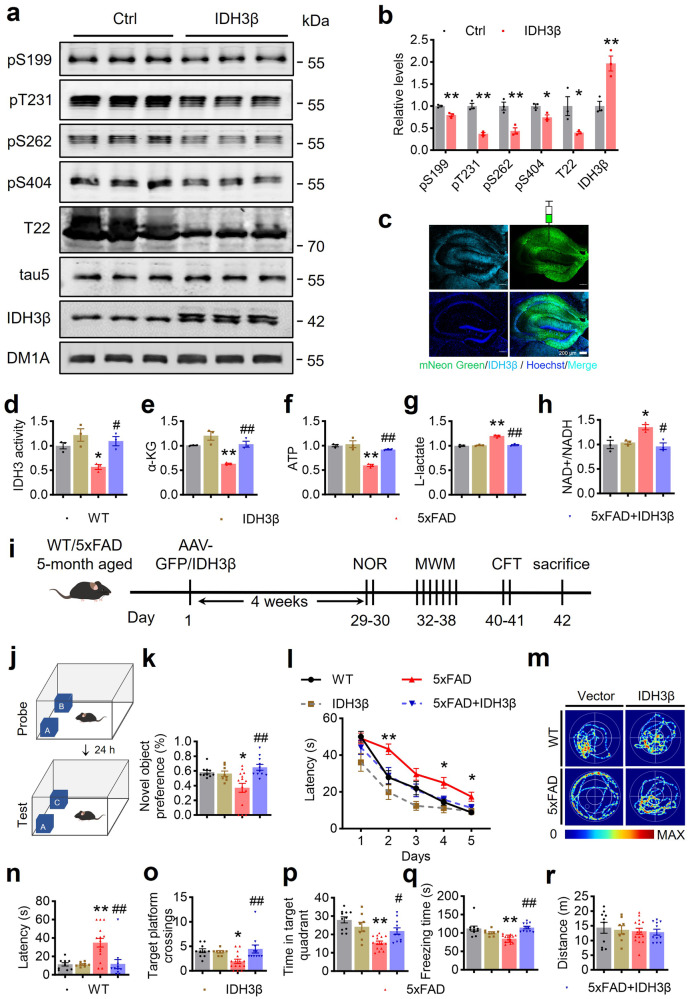
Upregulating IDH3β ameliorates learning and memory impairments and metabolic abnormalities in 5xFAD mice. **a**, **b** Upregulating IDH3β significantly decreased the phosphorylation levels of tau at S199 (**P = 0.006), T231 (**P < 0.001), S262 (**P = 0.008), S404 (*P = 0.023) and T22-positive tau (*P = 0.048) in HEK293 cells stably expressing tau. IDH3β, **P = 0.009; for each group (n = 3); Two-tailed Student’s t-test. **c** The eNeonGreen non-fusion AAV virus AAV-CMV-IDH3β-2A-eNeonGreen or the empty vector was infused stereotaxically into the bilateral hippocampus of 5-month-old 5xFAD or the wild-type control mice. The expression of IDH3β in the whole hippocampus was evidenced by fluorescence imaging. Scale bar, 200 µm. **d** The IDH3 activity was decreased in 6-month-old 5xFAD mice and exogenous expressing IDH3β restored the activity. *P = 0.036 *vs* WT, #*P* = 0.013 *vs* 5xFAD; for each group (n = 3); One-way ANOVA test followed by Tukey’s post hoc test. **e** The α-KG level was decreased in 5xFAD mice and exogenous expressing IDH3β restored α-KG to the normal level. **P = 0.006 *vs* WT, ##*P* = 0.004 *vs* 5xFAD; for each group (n = 3); One-way ANOVA test followed by Tukey’s post hoc test. **f** ATP level was reduced in 5xFAD mice and expressing IDH3β restored the ATP level. **P < 0.001 *vs* WT, ## *P* = 0.002 *vs* 5xFAD; for each group (n = 3); One-way ANOVA test followed by Tukey’s post hoc test. **g** The level of L-lactate was increased in 5xFAD mice and exogenous expressing IDH3β restored lactate level. **P < 0.001 *vs* WT, ##*P* < 0.001 *vs* 5xFAD; for each group (n = 3); One-way ANOVA test followed by Tukey’s post hoc test. **h** The ratio of NAD^ +^ /NADH increased in 5xFAD mice and expression of IDH3β restored the ratio. All assays were carried out by following the instructions of the commercially purchased assay kits. *P = 0.018 *vs* WT, #*P* = 0.010 *vs* 5xFAD; for each group (n = 3); One-way ANOVA test followed by Tukey’s post hoc test. **i** Schematics of the experiment procedure: One month after virus expression, the mice were examined by behavioral tests for learning and memory. NOR novel object recognition test, MWM Morris water maze test, CFT contextual fear condition test. **j**, **k** Upregulating IDH3β increased the preference for novelty in 5xFAD mice measured by NOR. *P = 0.016 *vs* WT, ##*P* < 0.001 *vs* 5xFAD; for each group (n = 8–15); One-way ANOVA test followed by Tukey’s post hoc test. **l**–**p** Upregulating IDH3β improved spatial learning and memory evidenced by decreased latency during 5-day training trials in MWM, and the decreased latency to the target platform, increased target platform crossings, and increased time spent in the target quadrant during probe test on day 7 in 5xFAD Mice. **l** Two-way repeated measures ANOVA test followed by Bonferroni’s post hoc test, day 2, **P = 0.008 *vs* WT, day 4, *P = 0.011 *vs* WT, day 5, *P = 0.011 *vs* WT; One-way ANOVA test followed by the Tukey’s post hoc test for (**n**–**p**), **n** **P < 0.001 *vs* WT, ##*P* < 0.001 *vs* 5xFAD; **o** *P = 0.032 *vs* WT, ##*P* = 0.006 *vs* 5xFAD; **p** **P < 0.001 *vs* WT, #*P* = 0.022 *vs* 5xFAD; for each group (n = 8–15). **q**, **r** Upregulating IDH3β improved contextual fear memory measured using CFT evidenced by the restored freezing time in 5xFAD mice. The mice did not show any difference in movement distance. **P < 0.001 *vs* WT, ##*P* < 0.001 *vs* 5xFAD; for each group (n = 8–15); One-way ANOVA test followed by Tukey’s post hoc test. The format of mean ± SEM was utilized to display the data

We elevated IDH3β expression in HEK293 cells that stably expressing tau to examine the potential for upregulating IDH3β to improve the pathologies and cognitive impairments linked to AD. Notably, the phosphorylation levels of tau at S199, T231, S262, S404, and T22-positive sites decreased significantly (Fig. 6a, b). Subsequently, the empty vector, or AAV-CMV-IDH3β-2A-eNeonGreen, was infused into the bilateral HPs of 5xFAD mice aged 5 months and confirmed the IDH3β expression in the HP through fluorescence imaging 1 month later (Fig. 6c). We also found that IDH3 activity was significantly restored in 5xFAD mice after IDH3β overexpression. Moreover, overexpressing IDH3β increased the levels of α-KG and ATP while decreasing the levels of L-lactate and the NAD^+^/NADH ratio in 6-month-old 5xFAD mice (Fig. 6d–h). Supplementing with α-KG also increased the level of ATP (Supplementary Fig. s7).

Next, a series of behavior tests were performed to assess cognitive capacity in 5xFAD mice with IDH3β overexpression (Fig. 6i). Upregulating IDH3β in 5xFAD mice led to increase the preference of novelty, as indicated using NOR (Fig. 6j, k). In 5-day training sessions in MWM, 5xFAD mice with upregulated IDH3β had a decreased latency in locating the hidden platform (Fig. 6l). Spatial memory improved as evidenced by reduced latency, more platform crossings, and longer time spent on the target quadrant following the upregulation of IDH3β in the probing test on day 7 (Fig. 6m–p). Upregulating IDH3β also improved contextual fear memory, as measured by the restored freezing time in 5xFAD mice (Fig. 6q). The mice did not show any change in their moving distance (Fig. 6r).

**Fig. 7:**
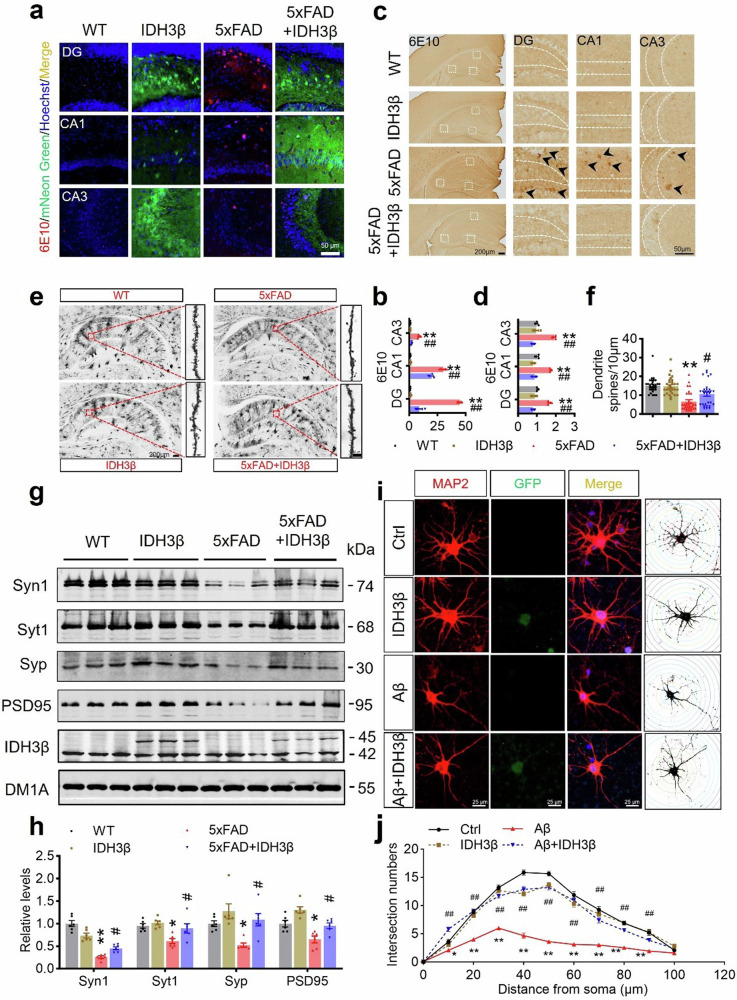
Upregulating IDH3β reduces Aβ plaques with improved dendrite/synapse plasticity. **a**, **b** Upregulating IDH3β reduced 6E10 (Aβ) staining in the hippocampal subsets of 5xFAD mice measured by immunofluorescence staining. Scale bar, 50 µm. **P < 0.001 *vs* WT, DG, and CA3, ##*P* < 0.001 *vs* 5xFAD, CA1, ##*P* = 0.002 *vs* 5xFAD; for each group (n = 3); One-way ANOVA test followed by Tukey’s post hoc test. **c**, **d** Upregulating IDH3β reduced 6E10 (Aβ) staining measured by immunohistochemistry in the hippocampal subsets of 5xFAD mice. Scale bar, 200 µm (hippocampus), 50 µm (area). **P < 0.001 *vs* WT, ##*P* < 0.001 *vs* 5xFAD; for each group (n = 3); One-way ANOVA test followed by Tukey’s post hoc test. **e**, **f** Upregulating IDH3β increased dendritic spines in the hippocampal neurons of 5xFAD mice measured by Golgi staining. Scale bar, 200 µm (hippocampus), 10 µm (spine). One-way ANOVA test followed by Tukey’s post hoc test, **P < 0.001 *vs* WT, #*P* = 0.015 *vs* 5xFAD, n = 30 neurons from three mice in each group. **g**, **h** Upregulating IDH3β restored expression levels of the synapse-associated proteins in the hippocampus of 5xFAD mice measured by Western blotting. Syn1, **P < 0.001 *vs* WT, #*P* = 0.036 *vs* 5xFAD; Syt1, *P = 0.010 *vs* WT, #*P* = 0.036 *vs* 5xFAD; Syp, *P = 0.036 *vs* WT, #*P* = 0.011 *vs* 5xFAD; PSD95, *P = 0.010 *vs* WT, #*P* = 0.029 *vs* 5xFAD; for each group (n = 6); One-way ANOVA test followed by Tukey’s post hoc test. **i**, **j** Upregulating IDH3β (lv-IDH3β-2A-GFP transfection 6 days and then Aβ treatment for 24 h) rescued Aβ-induced loss of neuronal complexity in primary neurons analyzed by Sholl analysis. Scale bar, 25 µm. One-way ANOVA test followed by Tukey’s post hoc test, 10 µm, *P = 0.021 *vs* Ctrl, ##*P* < 0.001 *vs* 5xFAD; 20–80 µm, **P < 0.001 *vs* Ctrl, ##*P* < 0.001 *vs* 5xFAD; 90 µm, **P < 0.001 *vs* Ctrl, ##*P* = 0.007 *vs* 5xFAD, n = 15 neurons from three mice for each group. The format of mean ± SEM was utilized to display the data

We further assessed the effect of upregulating IDH3β on AD-like pathologies in 5xFAD mice aged 6 months. 5xFAD mice had significantly more Aβ plaques in their hippocampal DG, CA1, and CA3 subsets compared to age- and sex-matched controls. However, upregulating IDH3β reduced Aβ plaques (6E10 staining) in all subregions of the hippocampus (Fig. 7a–d). Neuronal dendritic spines were reduced in 5xFAD mice while upregulating IDH3β restored the dendritic spines numbers to the normal level (Fig. 7e, f). Additionally, upregulating IDH3β restored the expression levels of synapse-associated proteins in 5xFAD mice, including synapsin1 (Syn1), synaptotagmin1 (Syt1), Syp, and PSD95 in 5xFAD mice (Fig. 7g, h). Moreover, overexpressing LV-IDH3β-2A-GFP in primary hippocampal neurons for 6 div rescued Aβ-induced loss of neuronal complexity, as analyzed by Sholl (Fig. 7i, j).

Collectively, our findings imply that stimulating IDH3β can boost synaptic plasticity, improve energy metabolism, and reverse AD-like pathologies in 5xFAD mice.

### Downregulating PAX6 ameliorates impaired memory and AD-like pathologies in 5xFAD mice

**Fig. 8:**
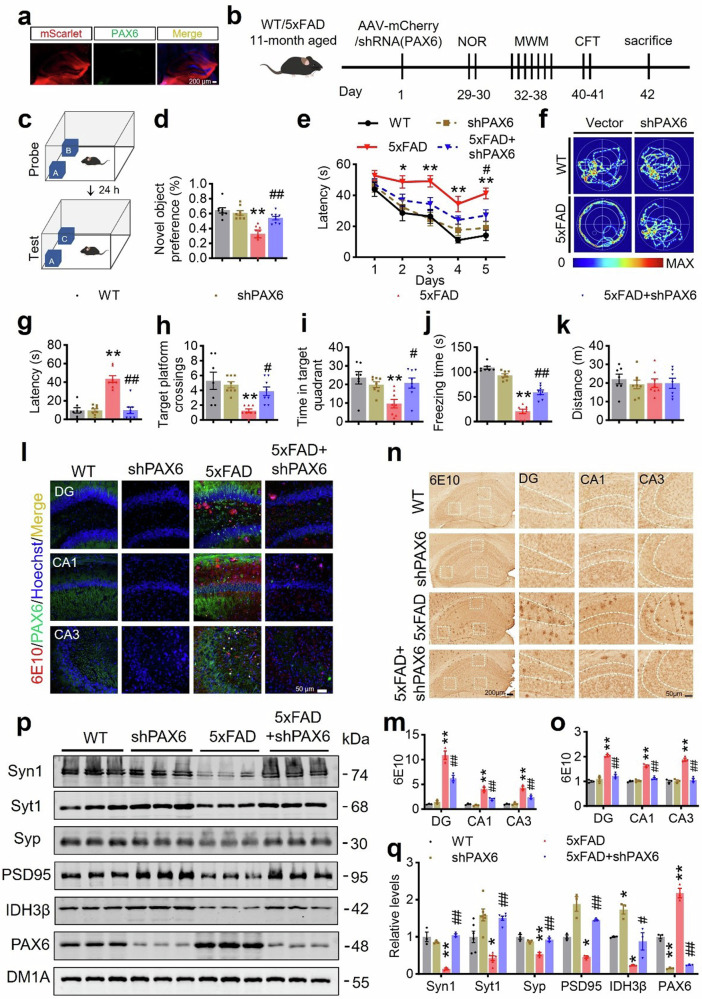
Downregulating PAX6 ameliorates memory deficits and AD-like pathologies in 5xFAD mice. **a**, **b** Schematics of the experiment procedure: the AAV virus AAV-U6-shRNA (PAX6)- CMV-mScarlet-WPRE or control shRNA AAV-U6-shRNA (NC2)-CMV-mScarlet-WPRE was infused stereotaxically into the bilateral hippocampus of 11-month-old 5xFAD or the wild-type mice. The expression of the virus in the whole hippocampus was evidenced by fluorescence imaging. Scale bar, 200 µm. After 1 month, the mice were examined by behavioral tests for learning and memory. NOR novel object recognition test, MWM Morris water maze test, CFT contextual fear condition test. **c**, **d** Downregulating PAX6 increased the preference for novelty in 5xFAD mice measured by NOR. **P < 0.001 *vs* WT, ##*P* = 0.002 *vs* 5xFAD; for each group (n = 8 - 9); One-way ANOVA test followed by Tukey’s post hoc test. **e**–**i** Downregulating PAX6 improved spatial learning and memory evidenced by decreased latency during 5-day training trials in MWM, and the decreased latency to the target platform, increased target platform crossings, and increased time spent in the target quadrant during probe test on day 7 in 5xFAD mice. **e** Two-way repeated measures ANOVA test followed by the Bonferroni’s post hoc test, day 2, *P = 0.022 *vs* WT, day 3, **P = 0.002 *vs* WT, day 4, **P < 0.001 *vs* WT, day 5, **P < 0.001 *vs* WT, #*P* = 0.042 *vs* 5xFAD; One-way ANOVA test followed by Tukey’s post hoc test for (**g**–**i**), **g** **P < 0.001 *vs* WT, ##*P* < 0.001 *vs* 5xFAD; **h** **P = 0.001 *vs* WT, #*P* = 0.036 *vs* 5xFAD; **i** **P = 0.003 *vs* WT, #*P* = 0.017 *vs* 5xFAD, n = 7–8 mice. **j**, **k** Downregulating PAX6 improved contextual fear memory measured by CFT evidenced by the restored freezing time in 5xFAD mice. The mice did not show any difference in movement distance. **P < 0.001 *vs* WT, ##*P* < 0.001 *vs* 5xFAD; for each group (n = 7–8); One-way ANOVA test followed by Tukey’s post hoc test. **l**, **m** Downregulating PAX6 reduced 6E10 (Aβ) staining in the hippocampal subsets of 5xFAD mice measured by immunofluorescence staining. Scale bar, 50 µm. DG, **P < 0.001 *vs* WT, ##*P* = 0.001 *vs* 5xFAD; CA1, **P < 0.001 *vs* WT, ##*P* < 0.001 *vs* 5xFAD; CA3, **P < 0.001 *vs* WT, ##*P* = 0.002 *vs* 5xFAD; for each group (n = 3); One-way ANOVA test followed by Tukey’s post hoc test. **n**, **o** Downregulating PAX6 reduced 6E10 (Aβ) staining measured by immunohistochemistry in the hippocampal subsets of 5xFAD mice. Scale bar, 200 µm (hippocampus), 50 µm (area). **P < 0.001 *vs* WT, ##*P* < 0.001 *vs* 5xFAD; for each group (n = 3); One-way ANOVA test followed by Tukey’s post hoc test. **p**, **q** Downregulating PAX6 restored expression levels of the synapse-associated proteins in the hippocampus of 5xFAD mice measured by Western blotting. Syt1, *P = 0.016 *vs* WT, ##*P* < 0.001 *vs* 5xFAD; for each group (n = 6). Syn1, **P < 0.001 *vs* WT, ##*P* < 0.001 *vs* 5xFAD, Syp, **P < 0.001 *vs* WT, ##*P* = 0.0014 *vs* 5xFAD, PSD95, *P = 0.023 *vs* WT, ## *P* < 0.001 *vs* 5xFAD, IDH3β, *P = 0.018, si-PAX6 vs WT, *P = 0.015, 5xFAD *vs* WT, #*P* = 0.037 *vs* 5xFAD; PAX6, **P < 0.001 *vs* WT, ##*P* < 0.001 *vs* 5xFAD; for each group (n = 3); One-way ANOVA test followed by Tukey’s post hoc test. The format of mean ± SEM was utilized to display the data

Considering the elevation of PAX6 during the AD process and its strong inhibition effect on IDH3β transcription, we next aim to test whether downregulating PAX6 could also alleviate AD pathology in 5xFAD mice. We delivered AAV-U6-shRNA (PAX6)-mScarlet or an empty vector to 11-month-old 5xFAD mice’s bilateral HP using stereotaxic infusion. After 1 month, we confirmed the expression of the virus in the HP through fluorescence imaging (Fig. 8a). Next, 12-month-old mice were examined for learning and memory (Fig. 8b). Downregulating PAX6 increased the preference for novelty in 5xFAD mice measured using NOR (Fig. 8c, d). Moreover, throughout the 5-day training trials, we recorded a reduced latency in MWM when locating the hidden platform (Fig. 8e). The probe test on day 7 revealed an improvement in spatial memory. This was verified by a decrease in latency to the target platform, an increase in the number of target platform crossings, and a longer time spent on the target quadrant (Fig. 8f–i). Additionally, downregulating PAX6 enhanced memory performance in the CFT, as indicated by the restored freezing time in 5xFAD mice (Fig. 8j). No statistically significant variations were observed in the distance covered by the groups (Fig. 8k).

We additionally validated the impact of PAX6 downregulation on AD-like pathologies in 5xFAD mice aged 12 months. Consistently, the number of Aβ plaques significantly increased in the hippocampal DG, CA1, and CA3 regions of 5xFAD mice compared to age- and sex-matched controls. Nevertheless, in the HP of 5xFAD mice, downregulating PAX6 decreased Aβ plaques (6E10 staining) (Fig. 8l–o). Additionally, we investigated the synapse-associated protein levels and found that downregulating PAX6 restored the expression of Syn1, Syt1, Syp, and PSD95 in 5xFAD mice (Fig. 8p, q).

Collectively, these findings indicate that downregulating PAX6 can also ameliorate memory impairments and AD-like pathologies in 5xFAD mice.

## Discussion

**Fig. 9:**
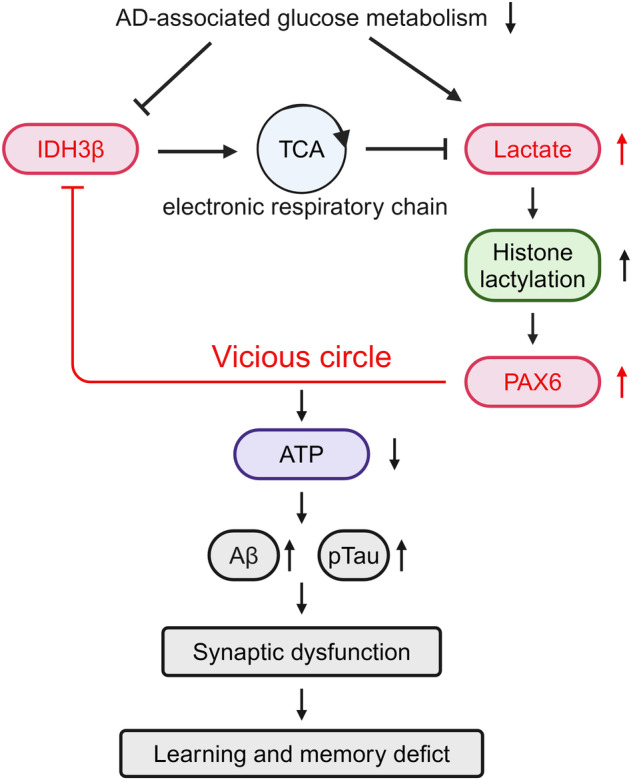
Working model showing how IDH3β-lactate-PAX6-IDH3β forms a positive feedback loop and how this vicious feedback loop drives the AD pathogeneses. Blocking the vicious feedback loop by upregulating IDH3β or downregulating PAX6 restores the homeostatic state and ameliorates AD pathology. La lactate. TCA tricarboxylic acid cycle

We found that both patients with AD and AD-transgenic mice had remarkably lower levels of IDH3β protein in our investigation. This reduction in IDH3β, along with impaired TCA and oxidation-phosphorylation coupling, resulted in intracellular lactate accumulation. This lactate accumulation, in turn, enhanced histone lactylation and PAX6 expression. Increased PAX6 further exacerbated the inhibition of IDH3β expression, creating a vicious positive feedback loop of IDH3β-lactate-PAX6-IDH3β. Ultimately, this feedback loop led to Aβ deposition, tau hyperphosphorylation, synaptic impairment, and learning memory dysfunction. By upregulating IDH3β and downregulating PAX6, it was possible to attenuate AD-like pathologies and cognitive deficits by improving cellular energy metabolism and decreasing histone lactylation (Fig. 9). Consequently, this study highlights the downregulation of IDH3β during the AD process and its crucial role in promoting AD. IDH3β may potentially function as a biomarker for the diagnosis of AD and drug screening, considering the notable reduction in levels observed in patients with AD.

Glucose metabolism is dramatically disrupted in AD. Both hyper- and hypo-metabolism have been reported in AD. A recent study by Naia et al. discovered mitochondrial hypermetabolism in the early stages of AD. This state is characterized by an elevation in oxidative phosphorylation, which is initiated by the functioning of mitochondrial complexes I, IV, and V. As a result, there is a higher risk of oxidative damage and Ca^2+^ overload.^10^ As the disease progresses, the brain transitions to a state of lower metabolic activity, accompanied by a decrease in the number of mitochondria in presynaptic terminals.^10^ An additional study involving individuals with amnestic mild cognitive impairment (aMCI) found that, during an 18-month clinical follow-up, those with aMCI who showed hypometabolism and low amyloid levels did not progress to AD, whereas those who showed amyloid-positive and hypometabolism did develop AD.^42^ As a compensatory mechanism for the early neuronal injury observed in the AD process, hypermetabolism may be involved, according to these results. Given that 5xFAD mice display indications of Aβ pathology at 1.5–2 months of age,^43^ the utilization of 6- or 12-month-old 5xFAD mice in our study suggests a more advanced stage of AD compared with younger mice. Consequently, our findings reveal hypometabolism in the present study.

The brain meets its energy requirements through adenosine triphosphate (ATP), which is generated via glycolysis and oxidative phosphorylation. Neurons in the brain exhibit higher levels of active pyruvate dehydrogenase and increased TCA cycle activity compared with astrocytes, thus favoring oxidative phosphorylation over aerobic glycolysis. Glycolysis contributes to 7% of total ATP production; however, mitochondria-driven oxidative phosphorylation supplies approximately 93% of the ATP utilized by neurons.^44–46^ IDH3 consists of three isoforms: IDH3α, IDH3β, and IDH3γ, and is crucial in the TCA cycle. In the HP, we found that IDH3α and IDH3γ did not change remarkably, while IDH3β decreased significantly in hippocampal neurons of both patients with AD an AD mouse model, highlighting the vulnerability of IDH3β in the AD process. Knockdown of IDH3β resulted in reduced levels of α-ketoglutarate (α-KG), an intermediate TCA cycle product, decreased ATP production, and an elevated NAD^+^ to NADH ratio. Conversely, overexpression of IDH3β remarkably mitigated these effects in 5xFAD mice. These findings suggest impaired oxidative phosphorylation during the progression of AD and underscore the critical involvement of reduced IDH3β in AD-like hypometabolism. IDH3β showed a notable decline with age, becoming statistically significant at 9 and 12 months in 5xFAD mice. However, IDH3β activity was significantly inhibited at 6 months in these mice. Based on the recovery of IDH3 activity following IDH3β overexpression in 5xFAD mice, we speculate that the inconsistency between protein level and activity may be due to method sensitivity. Therefore, our findings suggest that the combined detection of IDH3β protein level and activity may be more suitable for assessing AD progression.

The following underlying biological processes could be affected by histone modifications: 2-hydroxyisobutyrylation, butyrylation, methylation, crotonylation, acetylation, succinylation, malonylation, glutarylation, and β-hydroxybutyrylation. Most of these modifications involve metabolites.^25,27,28,47^ Recent research has demonstrated that lactate, an end product of glycolysis, controls gene transcription by acting as a post-translational modification of histones.^48,49^ In the present study, we also detected histone lactylation following IDH3β knockdown. Acetylation and lactylation target the same histone modification sites, including H4 at Lys12, Lys8, and H3 at Lys18. Consequently, an increase in lactylation would typically result in reduced acetylation at these sites. However, following IDH3β knockdown, we did not observe significant changes in histone acetylation at multiple sites. Considering the process of histone acetylation via acetyl-CoA in the presence of TCA slowdown,^32^ it is plausible that the reduction in IDH3β led to an increase in acetyl-CoA, thus counteracting the decrease in histone acetylation. Additionally, lysine acetylation is known to be highly dynamic,^32^ which may explain why histone lysine acetylation remains unchanged. Histone lactylation participates in multiple signaling pathways in normal and tumor tissues.^48,49^ We found that decreased IDH3β increased histone lactylation and then led to an increase in the expression of the transcription factor PAX6, which in turn inhibited the expression of IDH3β, forming a positive feedback loop that further suppressed IDH3β expression. We examined other top transcription factor binding sites in the IDH3β gene promoter and found no differences, suggesting that histone lactylation may not be a major regulator of other transcription factors.

IDH3β is expressed in various neural cells, including neurons, astrocytes, oligodendrocytes, and microglia.^50^ Both neurons and glial cells contribute to AD progression. For example, an imbalance between kinases and phosphatases within neurons increases tau hyperphosphorylation.^51^ Impairments in autophagy-lysosomal pathways and ubiquitin-proteasome systems within neurons and microglia cells reduce Aβ and tau pathology degradation.^52,53^ ATP production is crucial for these processes.^54,55^ It has been reported that insufficient energy metabolism and ATP supplementation lead to neurodegeneration,^56^ inhibit the phagocytic degradation function of microglia cells,^34^ and disrupt the neuroprotective effects of astrocytes.^57^ In the present study, we developed an IDH3β knockdown mouse model and observed AD-like tau hyperphosphorylation and synaptic protein loss in the HP. By injecting AAV-IDH3β with a CMV promoter, we overexpressed IDH3β in all neural cell types in the brains of 5xFAD mice. We found that this overexpression significantly improved learning and memory while restoring synapse-associated proteins and AD-like pathology in these mice. Although robust changes in IDH3β+ signals were detectable in pyramidal and granular cells of the hippocampus following IDH3β knockdown and overexpression, respectively, the effects of IDH3β manipulation on spatial memory, AD-like tau pathology, and synapse improvements should be attributed to the combined effect of IDH3β changes in all types of neural cells. Impaired glucose metabolism resulting from IDH3β reduction in all neural cells constitutes a significant contributing factor in accelerating AD-like pathologies. Although overexpressing IDH3β in all neural types restores cellular energy metabolism in the HP of 5xFAD mice, the same metabolic changes in neurons and glial cells might lead to different outcomes, especially in AD pathology. Therefore, further investigations on the manipulation of IDH3β in different types of neural cells are warranted and deserve attention in the future. Furthermore, IDH3 consists of two α catalytic subunits, a β structural subunit, and a γ metathesis subunit. It remains inactive in a monomeric state and only becomes fully active when a heterotetramer is formed in a 2:1:1 ratio. IDH3β promotes the assembly of heterotetramers. Although our data do not indicate that overexpression of IDH3β disrupts the holoenzyme, caution should be exercised regarding whether overexpression of IDH3β is the optimal strategy for preventing AD progression.

We also discovered that IDH3β expression is negatively regulated by PAX6. It’s interesting to note that in 6-month-old 5xFAD mice, we recorded a decrease in IDH3β protein levels, with a more pronounced decrease at 9 and 12 months. However, PAX6 showed a significant increase only in 12-month-old 5xFAD mice. These findings suggest that during the AD pathological process, a reduction in IDH3β may occur first, followed by an increase in lactate levels and elevated histone lactylation. This, in turn, promotes PAX6 expression and exacerbates the suppression of IDH3β expression. Thus, the cycle of AD pathology (i.e., Aβ and tau pathology) ➔ IDH3β reduction ➔ lactate increase ➔ PAX6 increase ➔ IDH3β reduction ➔ AD pathology may serve as a mechanism for the vicious cycle contributing to the relative later stages of AD.

In conclusion, our study demonstrates a significant decrease in IDH3β levels in AD patients and highlights the positive feedback inhibition loop of IDH3β-lactate-PAX6-IDH3β in accelerating AD progression.

## Materials and methods

### Human subjects

Human post-mortem cortical tissue sections were obtained from Guizhou Medical University, consisting of both patients diagnosed with AD and healthy control subjects. The additional details were presented in Supplementary Table s1. Subjects’ consent was acquired following the Declaration of Helsinki and authorized by the Tongji Medical School Ethics Committee.

### Animals

Male mice with homozygous IDH3β knockout exhibited infertility and an inability to produce mature spermatozoa, leading to an extended breeding cycle. Consequently, IDH3β knockdown heterozygotes were generated in C57BL/6 mice for this investigation. Shulaibao Biotechnology in Wuhan, China, supplied the male mice with either the wild-type (WT) or 5xFAD, which express the Florida (APPI716V) APP, London (APPV717I), and Swedish (APPK670N/M671L) mutations, in addition to the PS1L286V and PS1M146L mutations. The mice were either 5 or 11 months old. The Animal Care and Use Committee at Huazhong University of Science and Technology approved all procedures involving animals. These procedures were conducted in adherence to the regulations set forth by the Administration of Laboratory Animals in China. The mice were kept in a controlled laboratory environment with a temperature of 23 °C, unlimited access to food and water, and a 12-h cycle of light and dark. In every experiment, male mice weighing between 20 and 30 g were utilized. For immunostaining and Golgi staining, brain slices from 6-, 9-, and 12-month-old 5xFAD or age-matched WT mice were utilized; for Western blotting or metabolite assessments, brain tissue homogenate was utilized.

### Stereotactic injection

Mice were anesthetized with isoflurane, and recombinant adeno-associated virus (rAAV) rAAV-CMV-3xFLAG-P2A-mNeonGreen-tWPA (empty vector) and rAAV-CMV-IDH3β-3xFLAG-P2A-mNeonGreen-tWPA (IDH3β), as well as AAV-U6-shRNA (PAX6)-CMV-mScarlet-WPRE and AAV-U6-shRNA (NC2)-CMV-mScarlet-WPRE, were injected into the bilateral HP between the CA1 and DG subsets. From OBIO Technology (Shanghai, China), these viral vectors were obtained. The coordinates for the injection were as follows: −1.94 mm anterior-posterior, ±1.30 mm medial-lateral, and −1.75 mm dorsoventral from the bregma and dura assuming a flat cranium. The specific short hairpin RNA (shRNA) sequences targeting PAX6 were 5′-GAGTTTGAGAGGACCCATTAT-3′. Behavior assessments were performed on the mice 1 month later. After the behavioral evaluations, the mice were sacrificed for further biochemical analysis.

### Behavioral tests

The novel object recognition (NOR) test was conducted following previously established procedures.^58^ Mice underwent 5 min of handling per day for three consecutive days before the test. Placed in a box (50 × 50 × 50 cm) containing two objects positioned in the corner on the same side, mice were allowed 5 min of free exploration. After 24 hs, one of the objects was replaced, and mice were again given free exploration. Using a video tracking system (Sans, Jiangsu, China), the mice’s real-time location was monitored and their preference for the object within 3 cm of exploration was documented. The system, equipped with a top-mounted camera, recorded distance, time, and position. Before each test, both the box and objects underwent cleaning with 75% ethanol.

The Morris water maze (MWM) test was performed as described in a previous study.^59^ Mice underwent training in a circular pool, partitioned into four quadrants. The 120 cm in diameter and 60 cm high walls of the pool were outfitted with a camera video monitoring system (Taimeng, China) above the center to keep an eye on the mice’s whereabouts and speed. A week before the experiment, the experimental environment was provided for the mice to acclimate. The mice were allowed to swim freely after being introduced to the pool gently for 5 days. The latency was measured in seconds and the mouse was guided to remain on the platform for an additional 30 s if it could be detected within 60 s. A mouse was led to the platform and guided to remain there for 30 s if it was unable to locate it in 60 s. Mice were placed in various quadrants of the pool, excluding the quadrant containing the platform, and underwent three training sessions per day for 5 days. The interval between two consecutive training sessions for the same mice was at least 30 min.

On day 7, the platform was removed to assess spatial memory. The latency was quantified as the duration required for the mice to reach to the position on the platform. Within 60 s, the target platform crossing number was the number of times the mice traversed the platform area. The duration spent in the target quadrant constituted the time in target quadrant.

The contextual fear condition test (CFT) was performed as previously described.^60^ Mice were situated in a soundproof enclosure measuring 17 cm × 17 cm × 25 cm, featuring a floor capable of administering electric shocks. Time, distance, and motion trajectories were captured by a camera positioned above the center of the enclosure. The mice were allowed unrestricted movement within the enclosure for 3 min, following which three electric shocks of 2 s each (1 mA, with 1-min intervals) were administered. After a 24-h interval, the mice underwent testing for freezing time, defined as the duration when their displacement from the original position was less than 10%. The freezing time of the mice for 3 min was recorded using a video tracking system (Sans, China). Following each mouse’s assessment, the enclosure was cleaned with 75% alcohol.

### Immunohistochemistry and immunofluorescence

Immunohistochemistry (IHC) and immunofluorescence (IF) experiments were conducted following established protocols.^61^ Mice were anesthetized with isoflurane, fixed in an abdominal upward position, and intracardially perfused with 0.9% saline. Subsequently, brains were extracted and fixed in a 4% paraformaldehyde solution (PFA) in 0.01 M PBS at pH 7.4 for 24 h, followed by dehydration with 30% sucrose-PBS for a minimum of 48 h.

For IHC, brains were sectioned into 5 µm slices and paraffin-embedded. The dewaxing process included two 30-min treatments with xylene, followed by rehydration with ethanol at concentrations of 70%, 90%, and 95%. Antigen retrieval was performed by immersing the samples in a citric acid and sodium citrate solution (0.4 grams citric acid monohydrate, 2.64 grams sodium citrate dihydrate in one liter of ddH_2_O), boiled for 15 min. Subsequently, samples were treated with hydrogen peroxide for 30 min to eliminate endogenous hydrogen peroxidase. Following a 30-min blocking step with 5% bovine serum albumin/0.5% Triton X-100/PBS, the sections were subjected to an overnight incubation at 4 °C with primary antibodies. Subsequently, the sections were treated with secondary and third antibodies, respectively, for 1 h at 37 °C. Finally, samples were prepared utilizing a DAB peroxidase substrate kit (ZSGB-BIO), dehydrated through a series of alcohol concentrations (70%, 90%, and 95%), followed by two rounds of 10-min immersion in xylene, and sealed with neutral resin. Imaging was performed using a virtual slide microscope (Olympus SV120, Japan).

Brain sections were obtained for IF using a cryostat microtome (CM1900, Leica, Germany), with a dimension of 30 µm coronal sections. Following blocking for 30 min with 5% bovine serum albumin/0.5% Triton X-100/PBS, the sections were subjected to overnight incubation at 4 °C in the presence of primary antibodies, followed by incubation with secondary antibodies for 1 h at 37 °C and Hoechst for 10 min, then sealed with a mixture of 50% glycerol and 50% PBS. Slices were scanned using a confocal microscope (Carl Zeiss LSM800, Germany) for image acquisition. The steps for IHC on brains sliced with a cryostat microtome were the same as paraffin sectioning, except for the absence of antigen retrieval. The antibodies that were utilized are detailed in Supplementary Table s2.

### Western blotting

Western blotting was performed using an established in our laboratory.^4,62^ In summary, hippocampal tissue was homogenized in a buffer at a 1:10 ratio. Depending on the sample concentration, SDS-polyacrylamide gel electrophoresis (10% or 15% gel) was employed to separate proteins based on their molecular weight. Subsequently, the proteins of different molecular weights were transferred from the gel to nitrocellulose (NC) membranes using the wet transfer method. The primary antibodies were incubated at 4 °C for a whole night after the membranes were blocked with 5% skim milk. The next day, the membranes were cleaned and treated with Odyssey secondary antibodies. The images were captured using Odyssey (LI-COR Biosciences, USA) and quantified using ImageJ software. Supplementary Table s2 contains a list of the antibodies that were employed.

### Golgi staining

Utilizing the FD Rapid GolgiStain Kit (PK401, FD Neurotechnology) following the manufacturer’s guidelines, Golgi staining was performed. After anesthetizing the mice, their brains were immersed in an AB mixture (1:1) for one month. Throughout this period, the AB solution was refreshed on the first day and the mixture was agitated every 2 days, with the brains being shielded from light. Following that, the brains were immersed in solution C for 3–7 days, sectioned, and subjected to staining at a 100 µm thickness.

The staining procedure comprised the subsequent stages: a double wash in ddH_2_O for 4 min each, followed by a 10-min immersion in a solution mixture consisting of solution D, solution E, and ddH_2_O (in a 1:1:2 ratio), and then another double wash in ddH_2_O for the same duration. The samples were then dehydrated in 50%, 75%, and 95% alcohol gradients for 4 min each, with final dehydration in anhydrous ethanol for 4 min, and then in xylene for 3 rounds of 4 min each. After a waiting period of several hours, the samples were sealed, imaged using a light microscope (Nikon, Japan), and analyzed as previously described.^35,63^

### Cell culture and transfection

As previously described,^6^ 90% DMEM/Basic Glucose medium with 10% FBS was utilized to culture both mouse neuroblastoma N2a cells (N2a, RRID: CVCL_0470, Cat#: GDC0162, China Centre for Type Culture Collection) and human embryonic kidney 293 cells (HEK293, RRID: CVCL_0045, Cat#: GDC0067, China Centre for Type Culture Collection). The incubator was kept at 37 °C in a humidified atmosphere with 5% CO_2_. Tests for Mycoplasma on the validated N2a and HEK293 cell lines have yielded negative results. The Lipo-3000 RNA and DNA transfection kit (Cat#: L3000015, Thermo Fisher) was utilized to transfect cells with plasmids (pEGFP-MCS-2A-GFP, pEGFP-IDH3β-2A-GFP, pEGFP-PAX6-2A-GFP), or siRNAs, as directed by the manufacturer.

Primary hippocampal neurons were cultured as described previously.^64^ Neurons were obtained from Sprague Dawley rats (17 days embryos). The HP was removed, trypsinized at 37 °C for 10 min, dispersed equally with repeated blowing, filtered via a 0.45 µm filter, and cultured in 6- or 12-well plates with F-12 media containing 10% FBS. After 4 h, the F-12 medium was substituted with a 2 mL Neurobasal medium containing 2% B27 and 1% GlutaMAX. At 2 days in vitro (div), the neurons were infected with lentivirus (pcSLenti-CMV-IDH3β-3xFLAG-P2A-EGFP-WPRE and pcSLenti-CMV-3xFLAG-P2A-EGFP-WPRE), and 50% maintenance medium was renewed every 3 days. Cells were exposed to oligomeric Aβ after 6 days for 24 h. Cells that have been treated were analyzed using Western blotting analysis or IF.

### Reverse transcription and real-time quantitative PCR

In our laboratory, reverse transcription and real-time quantitative PCR were carried out following the established protocols.^3^ Total RNA was extracted using RNAisO PIus (Cat#: 9109, Takara), and then a reverse transcription reagent kit (Cat#: RR037A, Takara; Cat#: BL699A, Biosharp) was utilized to obtain cDNA. RT-PCR was carried out using a StepOnePlus Real-Time PCR Detection System (AB Applied Biosystems, Singapore). The PCR system is as follows: a blend of 1 µL cDNA, 1 µL forward and reverse primers, 7 µL RNase-free H_2_O, and 10 µL SYBR Green PCR master mixes (Cat#: Q711-02, Vazyme, China). GAPDH mRNA served as a reference for the mRNA levels of the relevant genes. Every experiment was conducted three times. The primer information was listed in Supplementary Table s3.

### Chromatin immunoprecipitation (ChIP) assay

The ChIP assay was performed using the ChIP kit (P2078, Beyotime) following the manufacturer’s instructions, following published methods.^64^ Using the DNA Purification Kit (D0033, Beyotime), purified DNA was produced. The quantification of DNA fragments in immunoprecipitated samples was carried out through quantitative real-time qPCR. Every experiment was conducted for a minimum of three times.

### Luciferase activity assay

The binding sequences of PAX6 to IDH3β gene promoter region, as well as their mutations, were synthesized and inserted into pGL3 luciferase reporter vectors. The Dual-Lumi Luciferase Assay Kit (RG088S, Beyotime) manufacturer’s protocol was followed throughout the experimental process. Utilizing the multifunctional enzyme marker Synergy 2 (BioTek, USA), relative luciferase activity was measured. At least three replications of each experiment were conducted. The binding sequences are listed in Supplementary Table s4.

### Metabolite assays

IDH3 activity was measured using the Isocitrate Dehydrogenase Assay Kit (Colorimetric) (ab102528, Abcam), while L-lactate levels were assessed with L-lactate Assay Kit (Colorimetric/Fluorometric) (ab65330, Abcam). The quantification of NAD^+^ and NADH was conducted using the NAD^+^/NADH Assay Kit (Colorimetric) (ab65348, Abcam). Alpha Ketoglutarate levels were determined with an Alpha-Ketoglutarate (alpha KG) Assay Kit (ab83431, Abcam). Additionally, ATP levels were measured utilizing the ATP Assay Kit (S0027, Beyotime). The Deproteinizing Sample Preparation Kit—TCA (ab204708, Abcam) was employed to remove proteins from the samples. Details of the reagents and resources utilized are provided in Supplementary Table s5.

### Statistical analyses

GraphPad Prism V9.0 (San Diego, CA, USA) was utilized for statistical analysis. These results are expressed as means ± SEM. The significance level was established at *P* < 0.05. Student’s t-test or ANOVA multiple comparisons test with Turkey or Bonferroni’s post hoc testing determined group differences.

### Supplementary information

Supplementary Materials

## Data Availability

The data that substantiate the results of this investigation are provided within the article. On request, the raw data utilized in the compilation of the figures can be obtained from the corresponding author. Gene expression profiles of brain regions can be accessed via the AlzData database (http://www.alzdata.org/). Transcription factor-binding information sources include GeneCards (https://www.genecards.org/) and JASPAR (https://jaspar.elixir.no/).
