# Correction: A positive feedback inhibition of isocitrate dehydrogenase 3β on paired-box gene 6 promotes Alzheimer-like pathology

**DOI:** 10.1038/s41392-024-01904-2

**Published:** 2024-07-15

**Authors:** Xin Wang, Qian Liu, Hai-tao Yu, Jia-zhao Xie, Jun-ning Zhao, Zhi-ting Fang, Min Qu, Yao Zhang, Ying Yang, Jian-Zhi Wang

**Affiliations:** 1 Department of Pathophysiology, School of Basic Medicine, Key Laboratory of Education Ministry of China/Hubei Province for Neurological Disorders, Tongji Medical College, Huazhong University of Science and Technology, Wuhan, China; 2 Department of Fundamental Medicine, Wuxi School of Medicine, Jiangnan University, Wuxi, Jiangsu 214122 China; 3 Hubei Provincial Key Laboratory for Applied Toxicology, Hubei Provincial Center for Disease Control and Prevention, Hubei Provincial Academy of Preventive Medicine, Wuhan, 430000 China; 4 Endocrine Department of Liyuan Hospital; Key Laboratory of Ministry of Education of China for Neurological Disorders, Tongji Medical College, Huazhong University of Science and Technology, Wuhan, 430077 China; 5 Co-innovation Center of Neuroregeneration, Nantong University, Nantong, 226000 China

**Keywords:** Diseases of the nervous system, Metabolic disorders

Correction to: *Signal Transduction and Targeted Therapy* 10.1038/s41392-024-01812-5, published online 29 April 2024

After online publication of the article^1^, the authors noticed that, owing to authors’ oversight in layer placement within the illustration, Golgi staining images representing both the 5xFAD group and the 5xFAD+IDH3β group were unintentionally duplicated in Fig. 7e; In Fig. 8b, 11-month-old mice were incorrectly written as 12-month-old; In Fig. 8c, Objects A and B were incorrectly labeled as A and A in the probe, while objects A and C were incorrectly labeled as A and B in the test; In Fig. 8f, WT was incorrectly written as Ctrl; In Fig. s7, *n* = 4 was incorrectly written as *n* = 3. The correct text and figures were provided as follows. The overall results and conclusions are not affected by the changes.
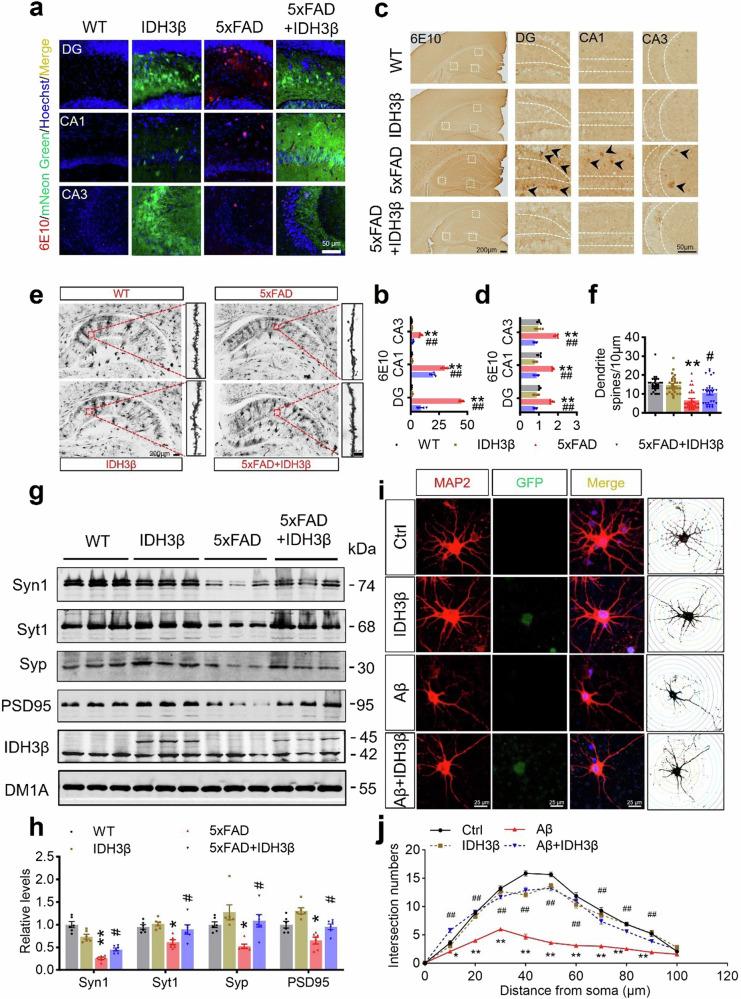

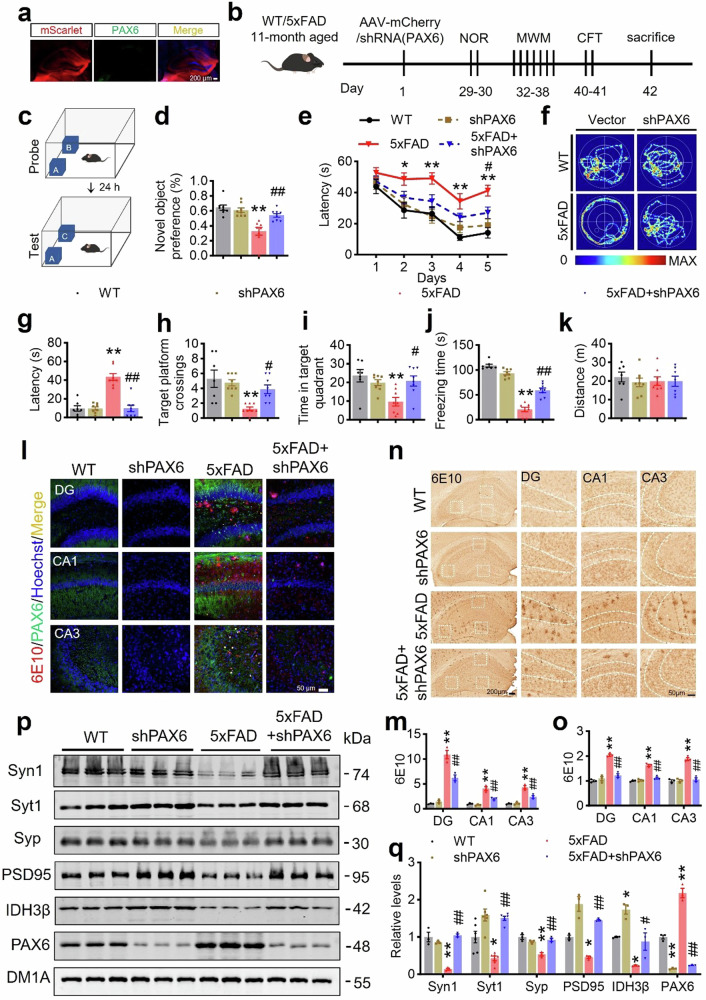


Fig. s7. DM-α-KG treatment increases ATP production.

DM-α-KG (7.5 mM for 24 h) treatment significantly increased ATP production in HEK293 cells stably expressing tau. For each group (*n* = 4), Two-tailed Student’s *t* test, **P < 0.001.

The original article has been corrected.
