# Supplementary material for: A positive feedback inhibition of isocitrate dehydrogenase 3β on paired-box gene 6 promotes Alzheimer-like pathology
Source: Signal Transduct Target Ther. 2024 Apr 29;9:105. doi: 10.1038/s41392-024-01812-5 (PMC11056379; doi:10.1038/s41392-024-01812-5)
Supplement: Supplementary file 1 — Supplementary Materials [file 41392_2024_1812_MOESM1_ESM.docx]

Supplementary Materials for

A positive feedback inhibition of isocitrate dehydrogenase 3β on paired-box gene 6 promotes Alzheimer-like pathology

Xin Wang, Qian Liu, Hai-tao Yu, Jia-zhao Xie, Jun-ning Zhao, Zhi-ting Fang, Min Qu, Yao Zhang#, Ying Yang#, Jian-Zhi Wang#

Correspondence to: Jian-Zhi Wang, E-mail: wangjz@mail.hust.edu.cn; Ying Yang, yingyang@hust.edu.cn; Yao Zhang, E-mail: zhangyaodoc@hust.edu.cn.

**This PDF file includes:**

Figures. s1 to s7

Tables s1 to s5


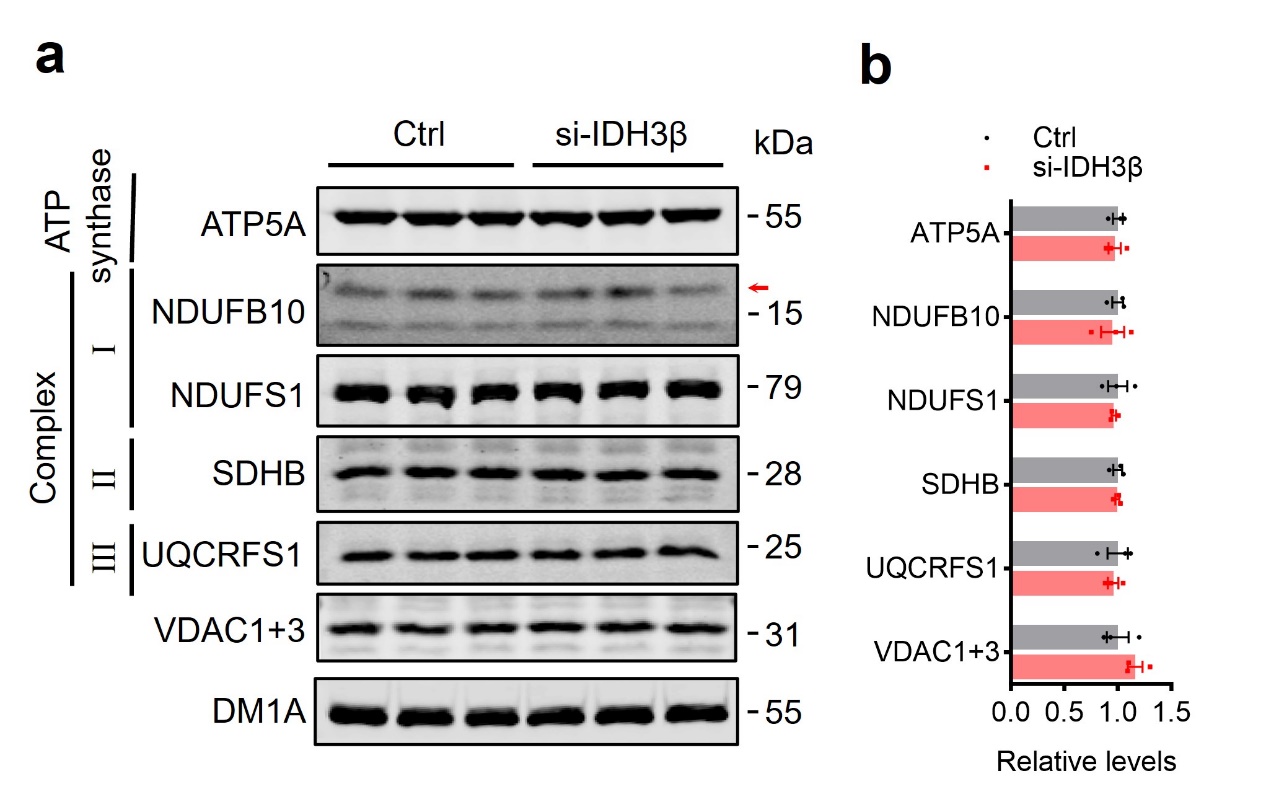


Fig. s1. Knockdown of IDH3β does not significantly affect several mitochondrial complex proteins.

(a, b) Knockdown of IDH3β did not affect NADUFB10 (the main band with an arrow) and NDUFS1 of respiratory chain complex I, SDHB of complex II, UQCRFS1/ RISP of complex III, α subunit of ATP synthase, and voltage-dependent anion channel, VDAC 1 and 3. For each group (n = 3), Two-tailed Student’s t-test. The format of mean ± SEM was utilised to display the data.


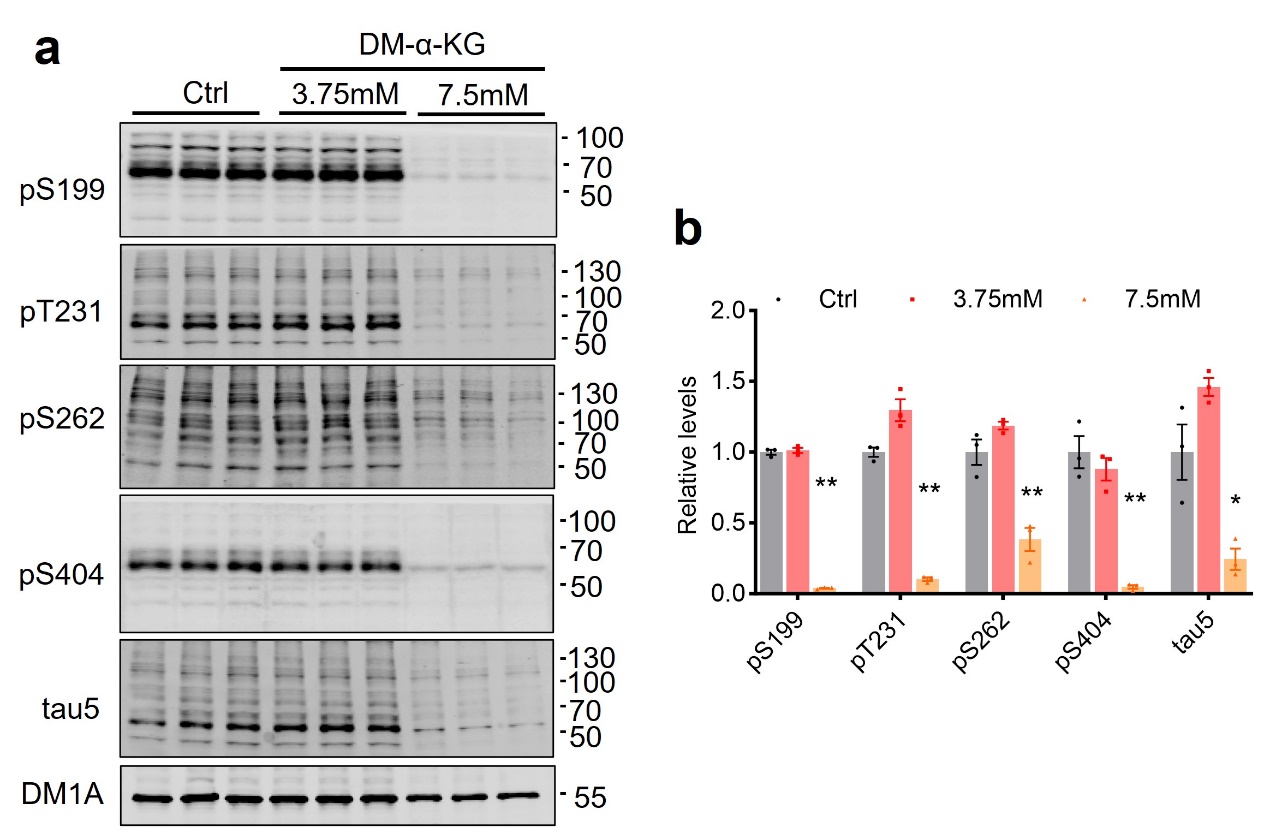


Fig. s2. DM-α-KG treatment decreases tau phosphorylation levels.

(a, b) DM-α-KG (7.5 mM for 24 h) treatment significantly decreased the phosphorylation levels of tau at S199 (the main band with an arrow) (***P* < 0.001), T231 (the main band with an arrow) (***P* < 0.001), S262 (the main band with an arrow) (***P* = 0.002), S404 (***P* < 0.001) and total tau (the main band with an arrow) (**P* = 0.013) in HEK293 cells stably expressing tau. One-way ANOVA test followed by Tukey’s post hoc test, for each group (n = 3), The format of mean ± SEM was utilised to display the data.


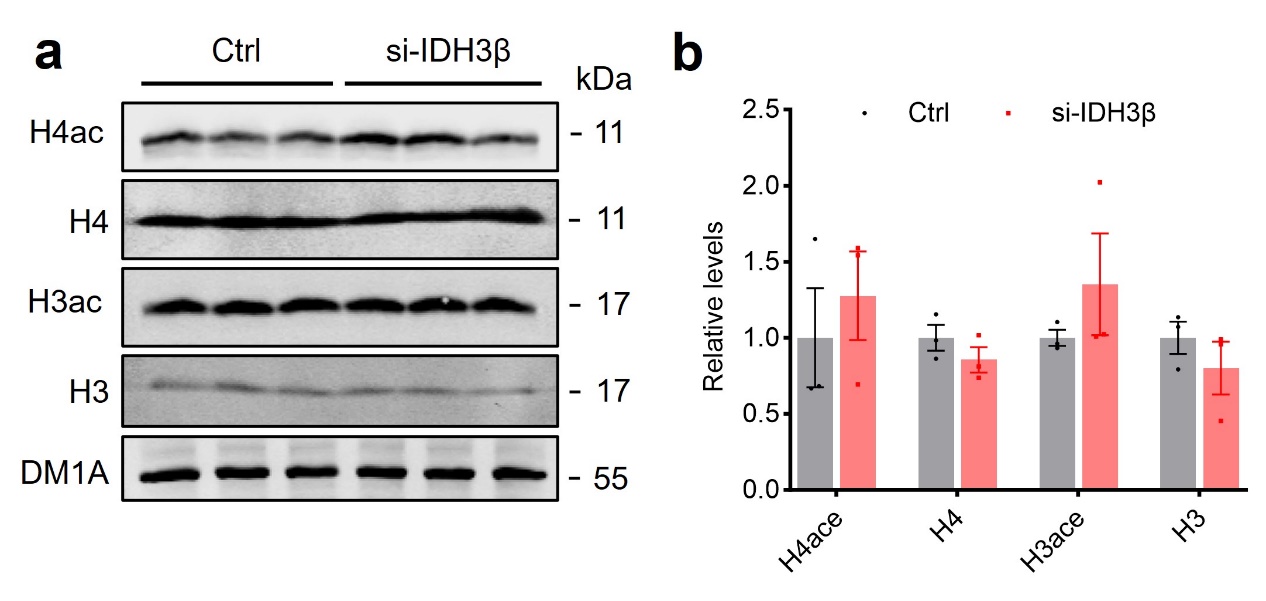


Fig. s3. Knockdown IDH3β does not affect histone acetylation.

(a, b) The acetylation levels of histone H3 at Lys9, Lys14, Lys18, Lys23, Lys27, and H4 at Lys5, Lys8, Lys12, and Lys16 sites were unchanged after knockdown of IDH3β in N2a cells. For each group (n = 3), Two-tailed Student’s t-test. The format of mean ± SEM was utilised to display the data.


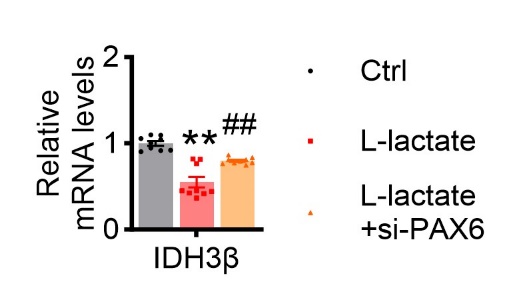


Fig. s4. L-lactate treatment decreases mRNA levels of IDH3β.

L-lactate (sodium lactate) (20 mM for 24 h) treatment significantly decreased mRNA levels of IDH3β, this decrease was completely reversed after PAX6 knockdown in N2a cells. One-way ANOVA test followed by Tukey’s post hoc test, ***P* < 0.001 vs Ctrl, ##*P* < 0.001 vs L-lactate, for each group (n = 9). The format of mean ± SEM was utilised to display the data.


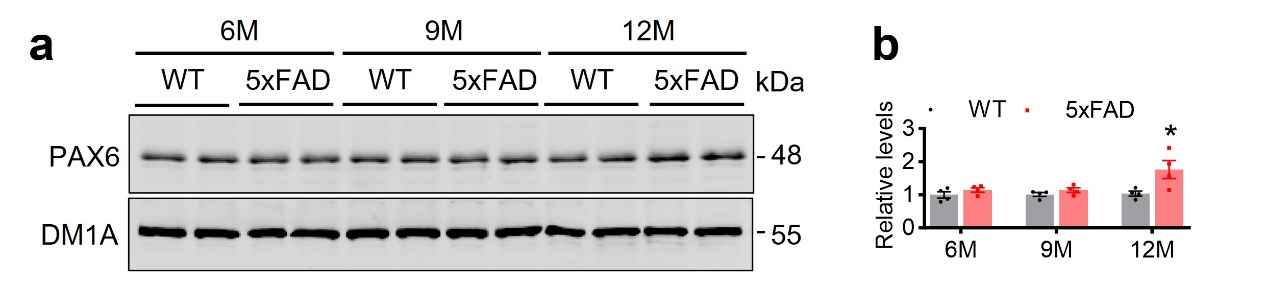


Fig. s5. PAX6 protein levels are increased in the hippocampus of 5xFAD mice.

(a, b) An age-dependent increase of PAX6 protein level in the hippocampus of 5xFAD mice was measured using Western blotting, while the increase was not detected in the littermates. For each group (n = 4), **P* = 0.04, Two-tailed Student’s t-test. The format of mean ± SEM was utilised to display the data.


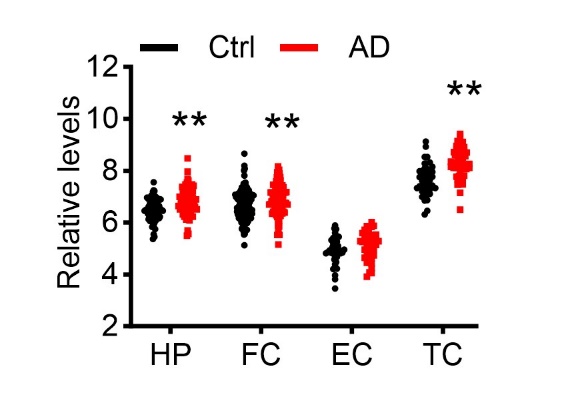


Fig. s6. PAX6 expression levels were increased in the AD patients.

PAX6 expression levels were significantly increased in the hippocampus (HP), frontal cortex (FC), entorhinal cortex (EC), and temporal cortex (TC) of AD patients based on the AlzData public database. HP and TC, ***P* < 0.001, FC, ***P* = 0.008.


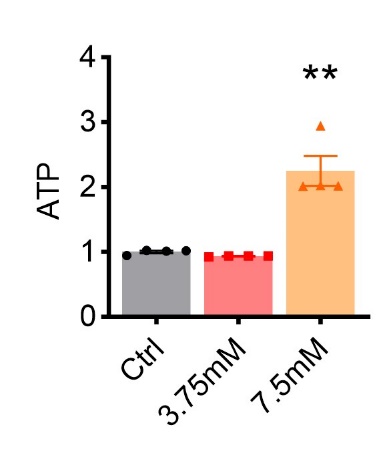


Fig. s7. DM-α-KG treatment increases ATP production.

DM-α-KG (7.5 mM for 24 h) treatment significantly decreased increased ATP production in HEK293 cells stably expressing tau. For each group (n = 4), Two-tailed Student’s t-test, ***P* < 0.001.

Table s1. Information of human brain sections

| #  ee | Type | Dementia degree | Age | Gender | PMI (h) |  |
| --- | --- | --- | --- | --- | --- | --- |
| 1 | HC | - | 63 | Female | 20 | Rheumatic polymyalgia |
| 2 | HC | - | 60 | Female | 32 | unknown |
| 3 | HC | - | 70 | Male | 29 | unknown |
| 4 | AD | +++ | 63 | Female | 72 | Acute myocardial ischemia |
| 5 | AD | +++ | 62 | Male | 48 | Organ failure |
| 6 | AD | +++ | 65 | Male | 12 | unknown |

Abbreviations: HC, Health Control. AD, Alzheimer’s desiease. PMI, postmortem interval. "-" means nondementia. "+", "++" and "+++" means low, medial and high level of dementia.

Table s2. The antibodies are as follows:

| Antibody | Source | Identifier |
| --- | --- | --- |
| Anti-IDH3β | Thermo Fisher | PA5-50336 |
| Anti-IDH3β | Abcam | ab247089 |
| Anti-IDH3β | ABclonal | A13742 |
| Anti-IDH3α | ABclonal | A14650 |
| Anti-IDH3γ | ABclonal | A13745 |
| Anti-PAX6 | ABclonal | A19099 |
| Anti-PAX6 | SAB | 49204 |
| Anti-PAX6 | CST | 60433 |
| Anti-MZF1 | ABclonal | A10356 |
| Anti-ZEB1 | ABclonal | A5600 |
| Anti-ZBTB6 | ABclonal | A15136 |
| Anti-NHLH1 | ABclonal | A15695 |
| Anti-NR3C1 | ABclonal | A19583 |
| PSD95 | ABclonal | A0131 |
| Synapsin1 (Syn1) | ABclonal | A17362 |
| Synaptophysin (Syp) | ABclonal | A6344 |
| Synaptotagmin1 (Syt1) | ABclonal | A0992 |
| Anti-ATPB | ABclonal | A11214 |
| Anti-Iba1 | Santa Cruz | sc-32725 |
| Anti-GFAP | Santa Cruz | sc-33673 |
| Anti-β-Amyloid, 1-16 (6E10) | Biolegend | SIG-39320 |
| Anti-Pan Kla | PTM BIO | PTM-1401 |
| Anti-H4K12la | PTM BIO | PTM-1411RM |
| Anti-H4K8la | PTM BIO | PTM-1415 |
| Anti-H3K18la | PTM BIO | PTM-1406RM |
| Anti-H3Kace | ABclonal | A17917 |
| Anti-H3 | ABclonal | A2348 |
| Anti-H4Kace | ABclonal | A22099 |
| Anti-H4 | ABclonal | A1131 |
| Anti-ATP5A | Abcam | Ab14748 |
| NDUFB10 | Abcam | ab196019 |
| NDUFS1 | Abcam | ab198955 |
| SDHB | Abcam | ab14714 |
| UQCRFS1/RISP | Abcam | ab14746 |
| VDAC1+3 | Abcam | ab14734 |
| Hoechst 33258 | Beyotime | C1011 |
| MAP2 | proteintech | 17490-1-AP |

Table s3. The primer information is as follows:

| Primer | Sequence (5’-3’) |
| --- | --- |
| GAPDH Forward | TTCCCGTTCAGCTCTGGG |
| GAPDH Reverse | CCCTGCATCCACTGGTGC |
| IDH3β Forward | AGCCGTCCATAAAGCCAACA |
| IDH3β Reverse | TCCCATGTCTCGAGTCCGTA |
| PAX6 Forward | CGAGAAGCGGCTTTGAGAAG |
| PAX6 Reverse | TCTACGATCTTCTGCCGGGT |
| IDH3β-ChIP1 Forward | AATACCAGGAACTCAGTACAG |
| IDH3β-ChIP1 Reverse | TCAATGCCGCCATGTTTC |
| IDH3β-ChIP2 Forward | TTGTAGAGTCGAGGTCTCC |
| IDH3β-ChIP2 Reverse | AGTTCAAGCGTGTAAGTGT |

Table s4. The binding sequences for Luciferase activity assays are as follows:

| Resource | Sequence (5’-3’) |
| --- | --- |
| IDH3β-ChIP1-ChIP2 | CCCTTTAGTCACACTTACACGCTTGAACTCATTTCTGACACTAGCGGGGCCGCGCCAGCTTCAGGCGTCACTTCCCACGCGACTTCCT |
| IDH3β-ChIP1 | CGGGGCCGCGCCAGCTTCAGGCGTCACTTCCCACGCGACTTCCT |
| IDH3β-ChIP2 | CCCTTTAGTCACACTTACACGCTTGAACTCATTTCTGACACTAG |
| IDH3β-ChIP1 mutation | CGGGGCCGCGCCAGCCCCACGCGACTTCCCCCACGCGACTTCCT |
| IDH3β-ChIP2 mutation | CCCTTTAGTCACACTCATTTCTGACACTACATTTCTGACACTAG |

Table s5. The reagent or resource are as follows:

| Reagent or resource | Source | Identifier |
| --- | --- | --- |
| Isocitrate Dehydrogenase Assay Kit (Colorimetric) | Abcam | ab102528 |
| L-Lactate Assay Kit (Colorimetric/Fluorometric) | Abcam | ab65330 |
| NAD/NADH Assay Kit (Colorimetric) | Abcam | ab65348 |
| Alpha Ketoglutarate (alpha KG) Assay Kit | Abcam | ab83431 |
| ChIP Assay Kit | Beyotime | P2078 |
| Dual-Lumi™ Luciferase Reporter Gene Assay Kit | Beyotime | RG088S |
| Enhanced ATP Assay Kit | Beyotime | S0027 |
| ChamQ Universal SYBR qPCR  Master Mix | Vazyme | Q711 |
| Deproteinizing Sample Preparation Kit - TCA | Abcam | ab204708 |
| DNA Purification Kit) | Beyotime | D0033 |
| IDH3β siRNA (m) | Santa Cruz | sc-62492 |
| Pax6 siRNA (m) | Santa Cruz | sc-36196 |
| Control siRNA | Santa Cruz | sc-81649 |
